# Preclinical evaluation of an ^18^F-labeled *N*^ε^-acryloyllysine piperazide for covalent targeting of transglutaminase 2

**DOI:** 10.1186/s41181-023-00231-1

**Published:** 2024-01-02

**Authors:** Robert Wodtke, Markus Laube, Sandra Hauser, Sebastian Meister, Friedrich-Alexander Ludwig, Steffen Fischer, Klaus Kopka, Jens Pietzsch, Reik Löser

**Affiliations:** 1 Helmholtz-Zentrum Dresden-Rossendorf, Institute of Radiopharmaceutical Cancer Research, Bautzner Landstraße 400, 01328 Dresden, Germany; 2 Helmholtz-Zentrum Dresden-Rossendorf, Institute of Radiopharmaceutical Cancer Research, Permoserstraße 15, 04318 Leipzig, Germany; 3 School of Science, Faculty of Chemistry and Food Chemistry, Technische Universität Dresden, Mommsenstraße 4, 01069 Dresden, Germany

**Keywords:** Biodistribution, Defluorination, Drug metabolism, Fluorine-18 chemistry, PET imaging, Pharmacokinetics, Plasma clearance

## Abstract

**Background:**

Transglutaminase 2 (TGase 2) is a multifunctional protein and has a prominent role in various (patho)physiological processes. In particular, its transamidase activity, which is rather latent under physiological conditions, gains importance in malignant cells. Thus, there is a great need of theranostic probes for targeting tumor-associated TGase 2, and targeted covalent inhibitors appear to be particularly attractive as vector molecules. Such an inhibitor, equipped with a radionuclide suitable for noninvasive imaging, would be supportive for answering the general question on the possibility for functional characterization of tumor-associated TGase 2. For this purpose, the recently developed ^18^F-labeled *N*^ε^-acryloyllysine piperazide **[**^**18**^**F]7b**, which is a potent and selective irreversible inhibitor of TGase 2, was subject to a detailed radiopharmacological characterization herein.

**Results:**

An alternative radiosynthesis of **[**^**18**^**F]7b** is presented, which demands less than 300 µg of the respective trimethylammonio precursor per synthesis and provides **[**^**18**^**F]7b** in good radiochemical yields (17 ± 7%) and high (radio)chemical purities (≥ 99%). Ex vivo biodistribution studies in healthy mice at 5 and 60 min *p.i.* revealed no permanent enrichment of ^18^F-activity in tissues with the exception of the bone tissue. In vivo pretreatment with ketoconazole and in vitro murine liver microsome studies complemented by mass spectrometric analysis demonstrated that bone uptake originates from metabolically released [^18^F]fluoride. Further metabolic transformations of **[**^**18**^**F]7b** include mono-hydroxylation and glucuronidation. Based on blood sampling data and liver microsome experiments, pharmacokinetic parameters such as plasma and intrinsic clearance were derived, which substantiated the apparently rapid distribution of **[**^**18**^**F]7b** in and elimination from the organisms. A TGase 2-mediated uptake of **[**^**18**^**F]7b** in different tumor cell lines could not be proven. Moreover, evaluation of **[**^**18**^**F]7b** in melanoma tumor xenograft models based on A375-hS100A4 (TGase 2 +) and MeWo (TGase 2 −) cells by ex vivo biodistribution and PET imaging studies were not indicative for a specific targeting.

**Conclusion:**

**[**^**18**^**F]7b** is a valuable radiometric tool to study TGase 2 in vitro under various conditions. However, its suitability for targeting tumor-associated TGase 2 is strongly limited due its unfavorable pharmacokinetic properties as demonstrated in rodents. Consequently, from a radiochemical perspective **[**^**18**^**F]7b** requires appropriate structural modifications to overcome these limitations.

**Supplementary Information:**

The online version contains supplementary material available at 10.1186/s41181-023-00231-1.

## Background

Activity-based probes (ABPs), consisting of a warhead for irreversible covalent bonding, a specificity-mediating moiety and a reporter group for robust and quantitative detection, are capable of the interrogative targeting of proteins, particularly enzymes, in a stable covalent manner with high specificity and selectivity in the complex living matter (Sadaghiani et al. 2007; Cravatt et al. 2008). The application of these molecular tools for biomedical purposes revolutionized the detection of enzymes in biological specimens, with regard to both ex vivo measurement as well as molecular imaging (Ou et al. 2018; Scott et al. 2021; Sotiropoulou et al. 2022). Regarding their application for molecular imaging, activity-based probes equipped with radionuclide-bearing moieties, in particular if labeling is carried out with radionuclides suitable for PET and SPECT imaging such as fluorine-18 and iodine-123, respectively, bear the potential for quantitative visualization of enzyme activity in vivo. This is because permanent radiotracer accumulation at sites of enzyme activity after clearance of the unbound probe can potentially account for high image contrast (Ren et al. 2011; Rotstein et al. 2014; Sawatzky et al. 2016; Withana et al. 2016; Rempel et al. 2017; Narayanaswami et al. 2019; Meyer and Braga 2021). In addition, imaging in the living organism can be complemented by ex vivo detection of the enzyme-radiotracer complex (Ren et al. 2011). However, a bottleneck for the successful translation of ABPs into agents for in vivo imaging are suitable pharmacokinetic properties, in particular sufficient metabolic stability and a favorable biodistribution in terms of rapid clearance from non-target tissues (Wyffels et al. 2010). The latter aspect is especially important if short-lived radionuclides such as fluorine-18 are employed for labeling (Greenwood et al. 2022). On the other hand, PET, apart from target-oriented imaging, is an excellent methodological tool for studying the pharmacokinetics of drugs and imaging agents non-invasively (Auberson 2016; Heller et al. 2018; Nerella et al. 2022; Wang et al. 2023).

**Scheme 1:**
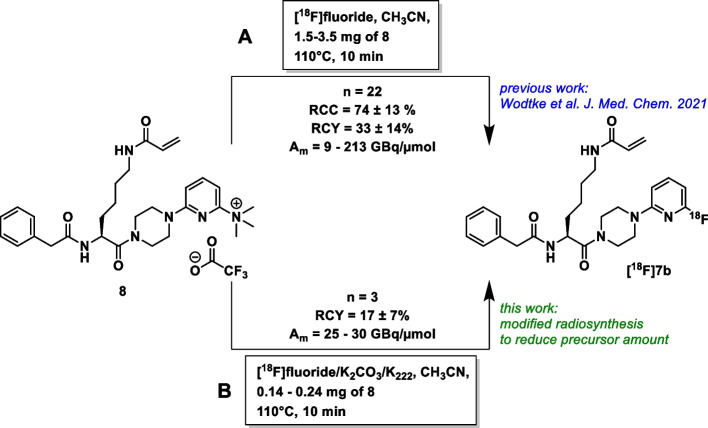
Radiosynthetic methods for [^18^F]7b used herein

An emerging target for theranostic approaches including molecular imaging is transglutaminase 2 (TGase 2) (Pietsch et al. 2013; van der Wildt et al. 2017a, b). This enzyme usually catalyzes Ca^2+^-dependent posttranslational modifications of proteins, e.g. by using glutamine and lysine residues to intra- and intermolecularly cross-link the proteins (Folk 1980; Griffin et al. 2002; Keillor et al. 2014). Its involvement in various pathophysiological processes including fibrosis (Elli et al. 2009; Olsen et al. 2011; Fell et al. 2021), celiac disease (Rauhavirta et al. 2019; Schuppan et al. 2021), and cancer (Eckert 2019; Tabolacci et al. 2019) stimulated the development of selective and potent inhibitors, especially targeted covalent inhibitors (Badarau et al. 2015; Büchold et al. 2022; Rangaswamy et al. 2022; McNeil et al. 2022; Mader et al. 2023; Cundy et al. 2023; Gates et al. 2023). Recently, we selected compound **7b** (numbering according to our previous publications), which is equipped with an acrylamide group as electrophilic warhead for reaction with the active site cysteine residue of TGase 2, for labeling with fluorine-18 due to its favorable parameters with regards to inhibitory activity, selectivity and physicochemical properties (Wodtke et al. 2018). The one-step radiosynthesis of **[**^**18**^**F]7b** (Scheme 1) by nucleophilic aromatic substitution of suitable precursor compounds proved to be challenging because of the precursor’s susceptibility to base-induced side reactions. A procedure for nucleophilic radiofluorination to furnish **[**^**18**^**F]7b** in a reliable manner was established using the trimethylammonio-substituted analog as precursor under minimalistic conditions without azeotropic drying by forming the ion pair of the cationic precursor and [^18^F]fluoride, and thus eluting of the latter from the QMA cartridge by the dissolved trifluoroacetate salt of the precursor. This accounted for obtaining **[**^**18**^**F]7b** in radiochemical yields of 33% and radiochemical purities > 97% (Wodtke et al. 2021). On this basis, it was possible to perform detailed radiopharmacological investigations in vitro and at the cellular level toward the characterization of this ABP. This confirmed the stability of the radiotracer in PBS and in the presence of GSH. Binding of **[**^**18**^**F]7b** to the target protein TGase 2 was analyzed by following the time-dependent formation of the enzyme-radiotracer complex with radio-TLC, which allowed for the determination of second-order rate constants that agreed well with the results of the enzyme activity assay. The binding of ligands such as Ca^2+^ ions and GTP-γS could be detected and analyzed very accurately using **[**^**18**^**F]7b** as a probe. At the cellular level, incubation of **[**^**18**^**F]7b** with lysates from tumor cells and subsequent separation by radio-SDS-PAGE allowed the sensitive determination of the TGase 2 protein present in the cells. **[**^**18**^**F]7b** also enabled the detection of TGase 2 in living cells, providing insight into the intracellular concentration of the transamidase-active protein for the first time, as demonstrated using human A375 melanoma cells. Furthermore, **[**^**18**^**F]7b** is capable of quantifying the expression in A375-derived xenograft tissue by radioluminographic ex vivo experiments on tissue sections. In addition, quantitative information on the expression levels of TGase 2 in different organs was obtained for the first time using this method, demonstrating the utility of the radiolabeled probe for the detection of this enzyme in various biological specimens (Wodtke et al. 2021).

These favorable results obtained in vitro and at the cellular level encouraged the investigation of **[**^**18**^**F]7b** for its radiopharmacological behavior in vivo, which is the objective of the study reported herein. To gain insight into the pharmacokinetic behavior at the organismic level, the biodistribution of **[**^**18**^**F]7b** was studied in NMRI nu/nu mice ex vivo and with small-animal PET imaging. This was complemented by ex vivo blood sampling and urine analysis using Wistar rats as model organism. Furthermore, the stability was assayed in vitro using liver microsomes. On this basis, solid pharmacokinetic parameters such as volume of distribution and plasma clearance were calculated. Finally, pilot experiments were performed toward imaging of the tumor-associated TGase 2 in a xenograft model derived from the human A375 melanoma cell line.

## Methods

### Radiosynthesis of [^18^F]7b

The radiosynthesis of **[**^**18**^**F]7b** under “minimalist” conditions (Richarz et al. 2014) using the respective trimethylammonio precursor **8** (Scheme 1) was recently described (Wodtke et al. 2021). To reduce the required precursor amount (≈ 3 mg for the “minimalist” conditions), a modified radiosynthetic method was developed based on our previously described microliter scale radiofluorination approach (Laube et al. 2021), which uses HPLC vials as reaction vessels. The aqueous [^18^F]fluoride (20–25 GBq) was adsorbed on an anion-exchange cartridge (QMA Plus Short from Waters preconditioned with 10 mL 1 M NaHCO_3_ and 10 mL of water) in a synthesizer and eluted with a solution (1 mL) of 22 mg of Kryptofix 222 and 1.0 mg of K_2_CO_3_ in 3% H_2_O/CH_3_CN. An aliquot (70–90 µL, 600–800 MBq) was transferred into a HPLC vial and was reduced to dryness at 90 °C under a helium stream. Subsequently, the trimethylammonio precursor dissolved in CH_3_CN (2 mg/mL, equal volume as the [^18^F]fluoride aliquot) was added and the mixture was heated at 110 °C for 10 min. The reaction mixture was diluted with 1 mL of water and purification was performed by semipreparative HPLC (LC-20A Prominence HPLC instrument by Shimadzu equipped with a gamma-detector LB 500 Herm). A Luna C18 5 µm column (Phenomenex, 250 × 10 mm) served as stationary phase. A binary gradient system of 0.1% trifluoroacetic acid in water (solvent A) and 0.1% trifluoroacetic acid in CH_3_CN (solvent B) served as the eluent. Gradient elution was performed using 30% eluent B for 3 min, 30% to 70% eluent B in 22 min, 70% to 95% eluent B in 1 min, 95% eluent B for 4 min, 95% to 30% eluent B in 1 min, and 30% eluent B for 9 min (total time of 40 min) at a flow rate of 5 mL/min. The peak representing the product was collected (approximately 5 mL) at t_R_ ≈ 15.0 min and immediately diluted with H_2_O to an overall volume of 25 mL. The resulting solution was subjected to solid-phase extraction by using a Chromafix C-18 ec (S) cartridge (preconditioned with 2 mL of ethanol and 10 mL of water). The cartridge was washed with water (1 × 2 mL) and the product was eluted with ethanol (1 × 1 mL). The product solution was evaporated to dryness and the residue was taken up in 0.5% ethanol/PBS (pH 7.4). An aliquot of that solution was withdrawn for analysis by radio-HPLC and radio-TLC. Radio-HPLC was performed with a Shimadzu Nexera X2 UHPLC system equipped with a gamma detector GABI from Elysia-Raytest GmbH. A C18 column Kinetex® from Phenomenex (5 µm, 100 Å, LC Column 250 × 4.6 mm) served as stationary phase. A binary gradient system of 0.1% trifluoroacetic acid in water (solvent A) and CH_3_CN (solvent B) at a flow rate of 1.0 mL/min was used as the eluent. Isocratic elution was performed using 38% eluent B for 10 min followed by washing of the column with 38% to 95% eluent B in 1 min, 95% eluent B for 5 min, 95% to 38% eluent B in 1 min, 38% eluent B for 5 min (total time of 22 min). Radio-TLC was carried out on Merck silica gel F-254 aluminium plates with ethyl acetate-acetone (1:1) as mobile phase. Radioactive spots were visualized using a CR-35 Bio scanner (Elysia-Raytest, Angleur, Belgium). The radioluminograms were analyzed by the software AIDA (Advance Image Data Analyzer, version 5.1 SP4 Build 1244).

### Liver microsome assay

Microsome experiments with **[**^**18**^**F]7b** in the presence of NADPH were performed according to the procedure recently described by us with slight modifications (Lemm et al. 2022). Incubations had a final volume of 250 µL. **[**^**18**^**F]7b** dissolved in 0.5% ethanol/PBS (100 µL; 0.2% ethanol final, e.g. 108 MBq/mL or 4.1 µM final) was mixed with PBS (112.5 µL) and murine liver microsomes (MLM, 12.5 µL of 20 mg/mL stock; 1 mg/mL final; Gibco™ Cat. No. MSMCPL, Lot. No. MS053-A) in a 1.5 mL Eppendorf tube and the mixture was warmed for 5 min at 37 °C. Subsequently, NADPH (25 µL of freshly prepared 20 mM solution in PBS, 2 mM final) was added and the mixture was incubated at 37 °C. As a control incubation, the NADPH solution was replaced by PBS. At distinct time points (5, 10, 15, 30, and 60 min), an aliquot (40 µL) was withdrawn and added to ice-cold CH_3_CN (160 µL). The mixture was vortexed for 30 s, stored on ice for 4 min and centrifuged (5 min at 16,100 × g). The resulting supernatant was used for radio-HPLC and radio-TLC analyses. For radio-HPLC, the Shimadzu Nexera X2 UHPLC system described above including the conditions for analysis was used. Radio-TLC was carried out on reversed-phase TLC plates (ALUGRAM RP-18W/UV254 from Macherey–Nagel, Düren, Germany) with a mobile phase of 40% CH_3_CN in water containing 0.1% trifluoroacetic acid. Visualization of radioactive spots and subsequent analysis was conducted as described above.

Testosterone (40 µM final) was used as positive control for the activity of the MLM and was separately treated and analyzed after incubation times of 5, 10, 15, 30, and 60 min as described above (0% ethanol final; HPLC gradient profile: 45% eluent B for 10 min followed by washing of the column with 45% to 95% eluent B in 1 min, 95% eluent B for 5 min, 95% to 45% eluent B in 1 min, 45% eluent B for 8 min; total time of 25 min). Typically, testosterone was completely consumed after 60 min, which served as indicator for a normal MLM activity.

For determining the *K*_m_ and V_max_ values of **7b** toward murine liver microsomes, in a set of experiments the degradation of **[**^**18**^**F]7b** at one defined activity concentration but at decreasing molar activities (and hence increasing concentrations of **7b)** was analyzed. For that, solutions of **[**^**18**^**F]7b** in 0.5% ethanol/PBS (10 µL; 0.1% ethanol final, 17 MBq/mL or 1.14 µM final) were mixed with the respective tenfold concentrated solutions of **7b** dissolved in 10% DMSO/PBS (5 µL, 1% DMSO final and 5/10/20/40/80 µM of **7b** final), PBS (27.5 µL) and murine liver microsomes (2.5 µL of 20 mg/mL stock) in a 1.5 mL Eppendorf tube and the mixture was warmed for 5 min at 37 °C. Subsequently, NADPH (5 µL of freshly prepared 20 mM solution in PBS, 2 mM final) was added and the mixture was incubated at 37 °C. At distinct time points (5, 10, 15, 20, and 30 min), an aliquot (5 µL) was withdrawn and added to ice-cold CH_3_CN (40 µL). The subsequent steps were performed as described above including analysis by radio-TLC.

Carrier-added samples (50 µM of **7b** and 1.8% DMSO final in MLM incubation) were stored at -20 °C and analyzed after radioactive decay by UPLC-MS/MS for structural elucidation of the metabolites. For UPLC-MS/MS, the following system was used: UPLC I-Class (Milford, Massachusetts, USA; binary gradient pump BSM, autosampler FTN, column manager CM, and diode array detector PDAeλ coupled to Waters Xevo TQ-S), column Aquity UPLC® BEH C18 column (waters, 100 × 2.1 mm, 1.7 µm, 130 Å), eluent: (A): 0.1% acetic acid in H_2_O (B): 0.1% acetic acid in CH_3_CN/CH_3_OH (1:1, v/v); flow rate 0.4 mL/min, gradient: 25% eluent B for 0.5 min, 25% to 75% eluent B in 5 min, 75% to 95% B in 0.5 min, 95% B for 1 min, 95% to 25% B in 1 min, 25% B for 0.5 min.; total time of 8.5 min, ESI + in MS, MS/MS, and MS/MS survey mode with the following MS parameters: capillary voltage 2.80 kV, cone voltage between 20 and 30 V, source temperature 150 °C, desolvation temperature 450 °C, collision gas (Ar) flow rate 0.15 mL/min, MS mode collision energy 12.00, MS/MS mode collision energy between 5 and 30 eV.

### Cell uptake studies

Binding and uptake of **[**^**18**^**F]7b** was investigated using the human melanoma cell lines A375, A375-hS100A4 and MeWo as well as NCI-H292 lung carcinoma and MDA-MB-231 mamma carcinoma cells. Cells were cultivated in Dulbecco’s modified Eagles’ medium supplemented with 10% heat-inactivated fetal calf serum (FCS), penicillin (100 U/mL), and streptomycin (100 µg/mL) at 37 °C and 5% CO_2_ in a humidified incubator. Uptake studies with **[**^**18**^**F]7b** were performed in confluent monolayer cultures in a 24-well plate format as described elsewhere with some modifications (Gassner et al. 2016; Laube et al. 2020). In brief, **[**^**18**^**F]7b** was added to the cells at an activity concentration of ≈ 0.5 MBq/mL (0.5 mL PBS per well) and cellular binding was investigated after 5, 10, 30, and 60 min at 37 °C. Additional uptake experiments with **[**^**18**^**F]7b** were performed in the presence of **7b** (10 µM, addition to cells 10 min prior to **[**^**18**^**F]7b**, blocking) and verapamil (100 µM, 10 mM stock in PBS, addition to cells 10 min prior to **[**^**18**^**F]7b**, blocking P-gp transport). The 24-well plates were placed on ice, the supernatant was removed and the cells were washed with ice-cold PBS (3 × 1 mL). Afterwards, the cells were treated with 0.5 mL NaOH (0.1 M containing 1% (w/v) sodium dodecylsulfate) for 30 min. Activity in cell lysates was measured with a Wizard™3’’ gamma counter. Protein concentration was determined using the BCA method.

Release of bound **[**^**18**^**F]7b** from cells under different conditions was followed after incubation of the cells with **[**^**18**^**F]7b** or **[**^**18**^**F]7b** + verapamil (100 µM) for 60 min as described above and subsequent removal of the supernatant followed by the addition of PBS (0.5 mL). After distinct time periods (5, 10, 30, 45, and 60 min), the 24-well plates were placed on ice, the supernatant was removed and the activity in the supernatant was measured with a Wizard™3’’ gamma counter. The cells were washed with ice-cold PBS (3 × 1 mL) and treated with 0.5 mL NaOH (0.1 M containing 1% (w/v) sodium dodecylsulfate) for 30 min. Activity in cell lysates was measured with a Wizard™3’’ gamma counter. Protein concentration was determined using the BCA method.

### Biodistribution in mice

Animal experiments were carried out according to the guidelines of the German Regulations for Animal Welfare. The protocols were approved by the local Ethical Committee for Animal Experiments (reference numbers 24–9168.11–4/2012–1 and 24.1–5131/449/49).

For biodistribution studies in normal mice, **[**^**18**^**F]7b** (0.57 ± 0.03 MBq corresponding to 27 ± 1 pmol in 0.2 mL 0.9% NaCl with a maximum of 10% ethanol (v/v)) was injected i.v. into nude mice (Rj:NMRI-Foxn1 nu/nu, Janvier Laboratories, Le Genest-Saint-Isle, France, body weight 27.7 ± 3.4 g) without (control conditions) or with co-injection of ketoconazole (3 mg/animal). A stock solution of ketoconazole (141 mM) was prepared in ethanol/Tween-80 (1:1, v/v) by shaking over night at 45 °C. This solution was diluted with 0.9% NaCl (1:4, v/v) prior injection. The animals were sacrificed at 5 and 60 min *p.i.* by CO_2_ inhalation and cervical dislocation. Organs and tissues of interest were excised, weighed, and radioactivity was determined using the Wizard™3’’ gamma counter. Activity in selected organs and tissues was expressed as % injected dose per weight (% ID/g) or % injected dose (% ID).

For biodistribution studies in tumor-bearing mice, a number of 5 × 10^6^ A375-hS100A4 or MeWo cells were resuspended in 100 µL PBS and injected subcutaneously into nude mice (Rj:NMRI-Foxn1 nu/nu). Tumor growth was monitored three times per week using caliper measurements. The injection of **[**^**18**^**F]7b** and subsequent steps were performed as described above.

### Small animal PET studies in mice

Small-animal positron emission tomography (PET) was performed using the nanoScan PET/CT scanner (Mediso Medical Imaging Systems, Budapest, Hungary). In case of tumor-bearing mice, PET imaging was conducted when tumors reached a size of 8 ± 3 mm. Tumor-bearing mice received between 5 and 10 MBq of **[**^**18**^**F]7b** delivered in 0.2 mL 0.9% NaCl with a maximum of 10% ethanol (v/v). The ketoconazole treated mice received 7.02 MBq of **[**^**18**^**F]7b** with 3 mg ketoconazole delivered in 0.25 mL 0.9% NaCl with 20% ethanol/Tween-80 (1:1, v/v). The respective control mouse received 5.15 MBq of **[**^**18**^**F]7b** delivered in the same solvent mixture but without ketoconazole. Administration of **[**^**18**^**F]7b** for all mice was done via intravenous injection through a tail vein catheter within the initial 30 s after scan start. General anesthesia was induced and maintained with inhalation of 10% desflurane in 30% oxygen/air (v/v). PET/CT scans were performed dynamically from 0 to 1 h after **[**^**18**^**F]7b** injection. Image recording, image reconstruction, and data analysis were performed as reported previously (Brandt et al. 2022). Standardized uptake values (SUV = [MBq detected activity/mL tissue]/[MBq injected activity/g body weight], g/mL) were determined in defined volumes of interest (VOIs) and reported as VOI-averaged SUV_mean_ ± range [min − max]. Assuming a density of 1 g/mL for body tissues, the SUVs become dimensionless.

### Metabolic stability and clearance in a normal wistar rat

The following procedure was performed as recently published (Laube et al. 2020). For assessing the in vivo stability, **[**^**18**^**F]7b** (69.8 MBq corresponding to 5.27 nmol in 0.5 mL 0.9% NaCl with 5% ethanol (v/v)) was injected *i.v.* into a male Wistar rat (body weight 170 g) under desflurane anesthesia (10% desflurane in 30% oxygen/air, v/v). Using a catheter, blood samples from femoral artery were taken at 1, 3, 5, 10, 20, 30, 60, and 120 min p.i. The resulting loss of volume was compensated by *i.v.* injection of E153. Plasma was separated by centrifugation (3 min; 13,000 × g) followed by precipitation of plasma proteins with ice-cold Supersol (EtOH 20% (v/v), Triton X-100 0.5% (v/v), EDTA 5 mM, *o*-Phenanthroline 0.5 mM, Saponin 0.1% (w/v)). Clear supernatant separated by a second centrifugation step (3 min; 13,000 × g) was analyzed by radio-HPLC (Hewlett Packard Series 1100 equipped with a γ-detector (Raytest Ramona), Zorbax SB-C18, 300 Å, 4 μm, 250 × 9.4 mm (Agilent), eluent A, 0.1% (v/v) trifluoroacetic acid in H_2_O; eluent B, 0.1% (v/v) trifluoroacetic acid in CH_3_CN). Gradient elution was performed using 95% eluent A for 5 min, 95% eluent A to 95% eluent B in 10 min, 95% eluent B for 5 min, and 95% eluent B to 95% eluent A in 5 min, 3 mL/min, 50 °C. The clear supernatant was also analyzed by radio-TLC using reversed-phase TLC plates (ALUGRAM RP-18W/UV254 from Macherey–Nagel, Düren, Germany) with a mobile phase of 50% CH_3_CN in water containing 0.1% trifluoroacetic acid. For total protein precipitation at 5 and 60 min *p.i.*, plasma samples were diluted with twice the volume of 15% trichloroacetic acid (TCA) in water instead of Supersol. Clear supernatant was separated by centrifugation (3 min; 13,000 × g) and analyzed as described above.

### Analysis of liver microsome data

For the t_1/2_ approach at n.c.a. level, the obtained fractions of intact **[**^**18**^**F]7b** (by radio-HPLC or radio-TLC analysis) were plotted against the time. Nonlinear regression was performed by one-phase decay (equation 1) as implemented in GraphPad Prism (GraphPad Prism 9.5.1.733), which provided the t_1/2_ values. The fraction at X = 0 min, Y_0_, was constrained to 1.0.

1 $${Y}_{0}=\left({Y}_{0}-Plateau\right)\times {e}^{-k\times X}+Plateau$$

Subsequently, the intrinsic clearance, CL_int_ (mL*min^−1^*kg^−1^), was calculated using equation 2 (Obach et al. 1997; Schneider et al. 2019)

2 $${CL}_{int}= \frac{ln2}{{t}_{1/2} \left(min\right)\times {fu}_{inc}}\times \frac{1}{\left[MLM\right](\frac{mg}{mL})}\times \frac{microsomal\; protein}{liver\; weight} \left(\frac{mg}{g}\right)\times \frac{liver\; weight}{body\; weight} \left(\frac{g}{kg}\right)$$

with the scaling factors of 60.1 mg/g (microsomal protein/liver weight for rats as no value for mice could be found) (Carlile et al. 1997) and 87.5 g/kg (liver weight/body weight for mice) (Davies and Morris 1993). The concentration of the MLMs was 1 mg/mL. The unbound fraction of **[**^**18**^**F]7b** in the murine liver microsomes, fu_inc_, was estimated to be 0.82 using equation 3 (Hallifax and Houston 2006)

3 $${fu}_{inc}=\frac{1}{1+[MLM]\times {10}^{0.072\times {logD}^{2}+0.067\times logD-1.126}}$$

with the logD_7.4_ value for **[**^**18**^**F]7b** of 2.1 (Wodtke et al. 2021) and the concentration of the murine liver microsomes, [MLM], of 1 mg/mL.

For the V_max_/K_m_ approach, the obtained fractions of intact **[**^**18**^**F]7b** (by radio-TLC analysis) were plotted against the time (fraction plot). The concentration plot was obtained by transformation of the fractions into concentrations of **7b** by taking the experimentally determined molar activity of **[**^**18**^**F]7b** into account (*i.e.* final concentration of 1.14, 6.16, 11.14, 21.14, 41.14, and 81.14 µM for **7b** in the performed experiments). Both plots were analyzed by nonlinear regression according to one-phase decay (equation 1). For the fraction plot, Y_0_ was constrained to 0.984 (fraction of intact **[**^**18**^**F]7b** after 30 min in the absence of NADPH) and Plateau to 0. Subsequently, the first derivative of this function at t = 0 min (equation 4) afforded the initial rates (v), which were multiplied with the respective total concentrations to provide v in µM/min.

4 $${Y}_{0}=\left({Y}_{0}-Plateau\right)\times k$$

The obtained v values were then plotted against the total concentrations of **7b** and the respective curves were analyzed by nonlinear regression using the Michaelis–Menten equation 5 with [S] being the total concentrations.

5 $$v=\frac{{V}_{max}\times [S]}{{K}_{m} + [S]}$$

CL_int_ (mL*min^−1^*kg^−1^) was calculated using equation 6 (Obach et al. 1997) and the same values for the scale factors, [MLM] and fu_inc_ as described for equation 2.

6 $${CL}_{int}= \frac{{V}_{max} (\frac{\mu M}{min})}{{K}_{m}\left(\mu M\right)\times {fu}_{inc}}\times \frac{1}{\left[MLM\right](\frac{mg}{mL})}\times \frac{microsomal\; protein}{liver\; weight} \left(\frac{mg}{g}\right)\times \frac{liver\; weight}{body weight} \left(\frac{g}{kg}\right)$$

### Analysis of blood clearance data

The measured activity concentrations (decay-corrected) in the blood samples of the Wistar rat were transformed into concentrations of **7b** by taking the molar activity of **[**^**18**^**F]7b** into account. These values were corrected by the fractions of intact **[**^**18**^**F]7b** as analyzed by radio-HPLC or radio-TLC. These corrected concentrations were plotted against the time and analysis was performed by nonlinear regression according to two-phase decay as implemented in GraphPad Prism (Plateau was set to 0, Fig. 3B). However, for derivation of pharmacokinetic parameters, the corrected concentrations were converted to their log values and plotted against the time (semi-logarithmic plot, see inset in Fig. 3B). By linear regression of the data from 3 to 30 min, values for c_02_ (putative concentration of **7b** at the beginning of the elimination phase) and *k*_el_ (elimination rate constant) were derived. C_02_ was obtained as antilog of the intercept at the y-axis and *k*_el_ represents the slope of the line. By knowing these two parameters as well as the applied dose (D = 5.27 nmol), the apparent volume of distribution (V_D_) and the plasma clearance (CL) was calculated using equations 7 and 8 (Freissmuth et al. 2016).

7 $${V}_{D}=\frac{D}{{c}_{02}}$$

8 $$CL={k}_{\beta }\times {V}_{D}$$

## Results

### Radiosynthesis of [^18^F]7b

The alternative radiosynthesis of **[**^**18**^**F]7b** is shown in Scheme 1 (B) together with the original strategy (A), which was recently published by us (Wodtke et al. 2021). The motivation to change the radiosynthetic route originates from the high precursor amount (≈3 mg in methanol) necessary for efficient elution of [^18^F]fluoride from the QMA cartridge in the absence of base and cryptand Kryptofix 222. By application of our recently published microliter scale radiofluorination approach (Laube et al. 2021) and elution of [^18^F]fluoride with a modified solution of Kryptofix 222 (22 mg) and K_2_CO_3_ (1 mg) in 3% H_2_O/CH_3_CN (1 mL), radiofluorination was conducted in a volume of less than 100 µL with 500–800 MBq of [^18^F]fluoride and less than 0.3 mg of precursor **8**. Compound **[**^**18**^**F]7b** was the main radiolabeled product, which was, however, accompanied by the formation of various radiolabeled side-products of lower activity (Additional file 1: Fig. S1). After HPLC purification and solid-phase extraction, 40–150 MBq of **[**^**18**^**F]7b** were obtained in 0.5% EtOH/PBS at activity concentrations of > 200 MBq/mL and radiochemical and chemical purities of ≥ 98% (Additional file 1: Fig. S2). The total radiosynthetic procedure was finished in less than 60 min. **[**^**18**^**F]7b** obtained by this radiosynthetic procedure was used for the MLM studies, while **[**^**18**^**F]7b** accessed by our previous base-free method was used for all other investigations herein.

#### Biodistribution and PET imaging in normal mice

**Fig. 1:**
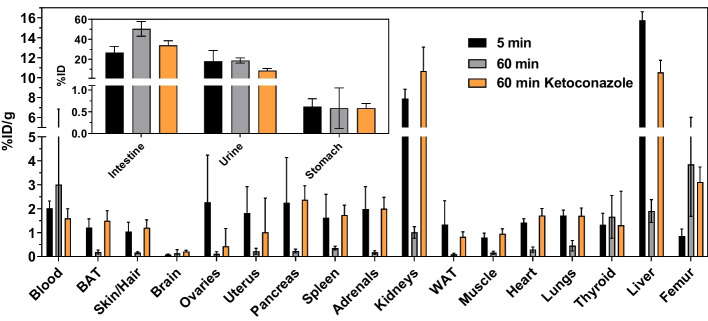
Biodistribution of** [**^**18**^**F]7b** in normal mice. Activity uptake in distinct organs is expressed as percentage of injected dose per organ mass (%ID/g) or as percentage of injected dose (%ID; inset). Ketoconazole (3 mg/animal) was co-injected with **[**^**18**^**F]7b**. Data shown are mean values (± SD) of 4 (for 5 min and 60 min ketoconazole) and 8 (for 60 min) mice which received a single injection of **[**^**18**^**F]7b** (≈0.5 MBq/animal). BAT and WAT are abbreviations for brown and white adipose tissue, respectively

**Fig. 2:**
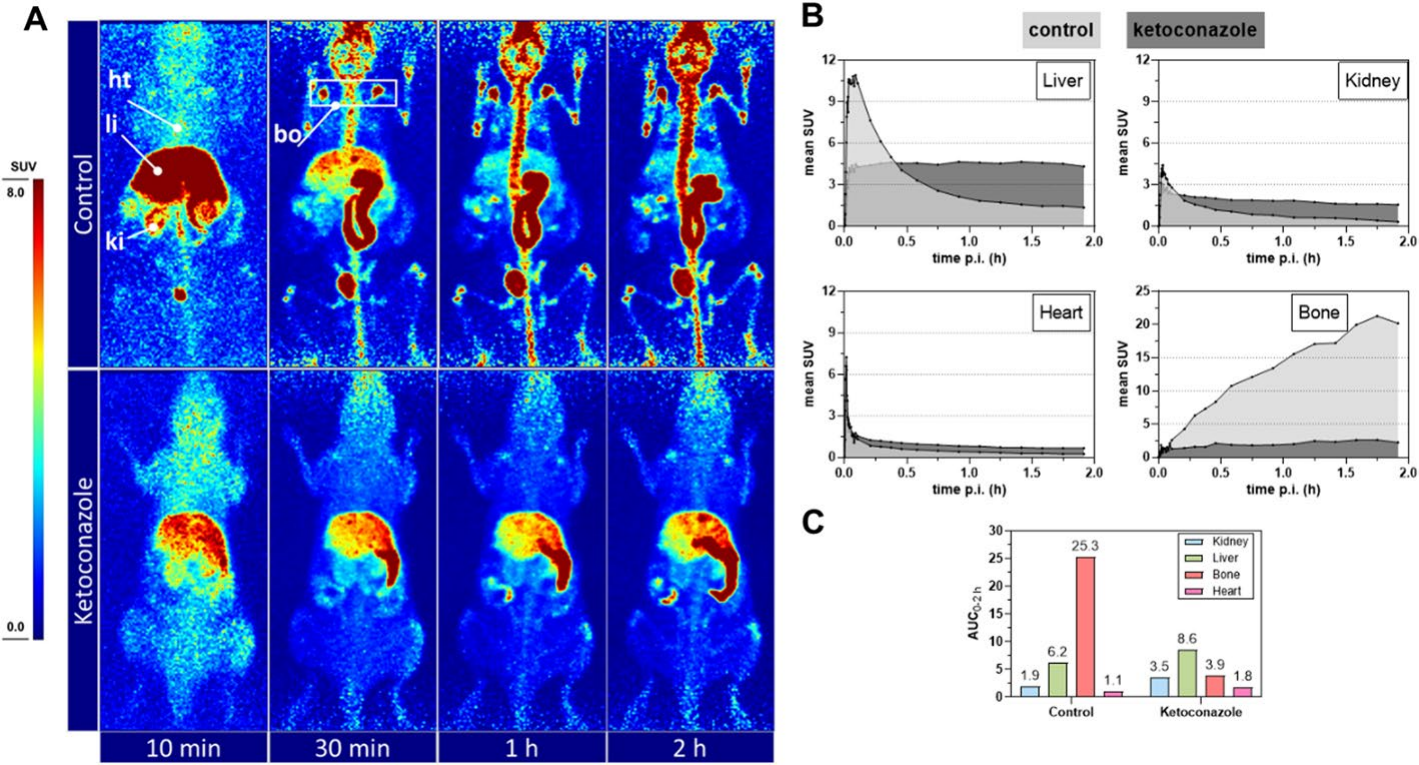
PET imaging for **[**^**18**^**F]7b** in normal mice. **A** PET images at selected time points after intravenous injection of **[**^**18**^**F]7b** in normal mice under control conditions (upper panel, 5.16 MBq) and with co-injection of ketoconazole (lower panel, 7.02 MBq and 3 mg ketoconazole). Images are presented as maximum intensity projections and shown at a common scale (SUV 0–8). Indicated time points correspond to the following time frames: 5 min [4 − 6 min], 30 min [25 − 30 min], 1 h [50 − 60 min], and 2 h [110–120 min]. Anatomical positions of liver (li), kidney (ki), bone (bo), and heart (ht) used for SUV quantification are exemplarily shown for the control mouse. **B** Standard uptake values (SUV, decay-corrected) for control mouse and ketoconazole treated mouse as a function of time (up to 2 h, in dynamic mode) obtained by PET acquisition (same experiments as in** A**). Data shown are for one mouse each. For a better orientation, dotted lines at SUVs of 3, 6, and 9 (for liver, kidney and heart) and 5, 10, and 15 (for bone) were drawn. **C** Summary of calculated AUC_0-2 h_ values for the curves shown in** B**

For ex vivo biodistribution of **[**^**18**^**F]7b**, normal mice were injected with ≈0.5 MBq of the radiotracer and activity distribution in organs of interest was measured at 5 and 60 min *p.i.* (Fig. 1). There seemed to be no activity enrichment for **[**^**18**^**F]7b** and its potential metabolites as the activity uptake decreases from 5 to 60 min *p.i.* for almost every organ. The highest activity uptake at 5 min *p.i.* can be observed for the liver (15.7%ID/g) followed by the kidneys (7.9%ID/g). Exceptions with regards to the declining activity over time were visible for the thyroid and the femur bone tissue. While the activity uptake in the thyroid at 60 min *p.i.* was comparable to that at 5 min *p.i.*, the uptake in the femur bone tissue was significantly higher. Moreover, activity uptake in the femur at 60 min *p.i.* was the highest among all organs (3.9%ID/g). Regarding the excretion, the majority of injected activity was found in the intestinal content (50%ID) and a significantly lower percentage in the urine (19%ID) at 60 min *p.i.* (Fig. 1 inset), which is in line with the higher activity uptake observed in the liver compared to the kidneys. PET imaging studies in normal mice over 1 h largely confirmed the biodistribution results, in particular the activity uptake in the bone tissue as e.g. the spinal column, skull, joints and epiphyses were visible with high contrast (Fig. 2A). As the activity uptake in bone tissue originated most likely from metabolically released [^18^F]fluoride, biodistribution and PET imaging were also conducted in mice pretreated with ketoconazole, which is a potent inhibitor of CYP3A enzymes (Greenblatt et al. 2011). These investigations demonstrated that pretreatment with ketoconazole significantly reduced the uptake of ^18^F-activity in the aforementioned parts of the skeleton (Figs. 1 and 2B). Furthermore, the activity uptake was increased in almost all organs in the presence of ketoconazole compared to animals without pretreatment by this compound. In contrast, the amount of activity in urine and intestinal content was lowered at 60 min *p.i.* in the presence of ketoconazole.

### In vivo* and *in vitro* metabolism*

**Fig. 3:**
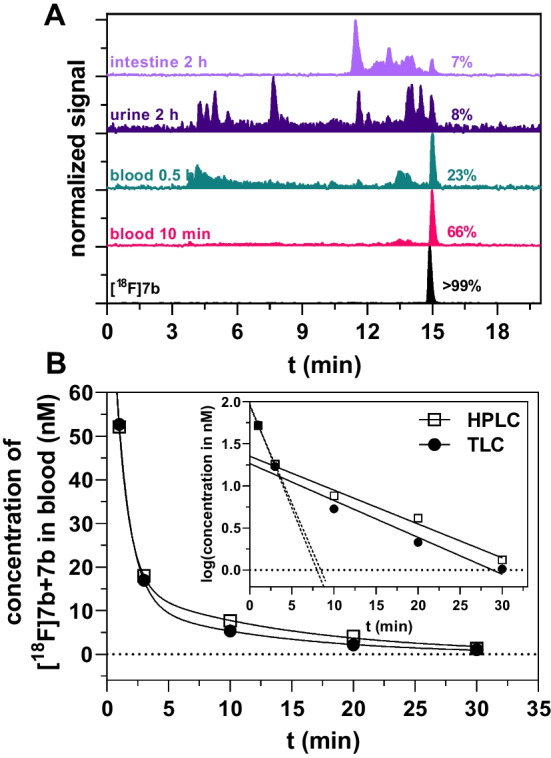
Metabolic stability and arterial blood clearance of **[**^**18**^**F]7b** in a normal Wistar rat. **A** Radio-HPLC chromatograms of **[**^**18**^**F]7b** in samples of blood and excretion media taken at different time points after i.v. injection of **[**^**18**^**F]7b**. **B** Time course of arterial blood clearance of intact **[**^**18**^**F]7b** as calculated from activity measurement of blood samples and fraction of intact **[**^**18**^**F]7b** determined by radio-HPLC and radio-TLC (Additional file 1: Fig. S3) including nonlinear regression according to two-phase decay. The inset shows the respective semi-logarithmic plot with separate linear regressions for the time points 1–3 min (dotted line, extrapolated up to the x-axis) and 3–30 min (solid line, extrapolated up to the y-axis). From the results of the latter linear regression, c_02_ and *k*_el_ were derived (see Methods section). Data in **A** and **B** are from the same experiment, in which **[**^**18**^**F]7b** (69.8 MBq, 5.27 nmol) was injected into a healthy Wistar rat (body weight of 170 g)

To obtain insight into the in vivo metabolism of **[**^**18**^**F]7b**, its fate was monitored after injection into a Wistar rat by analyzing blood samples and excretion media through radio-TLC and radio-HPLC (Fig. 3 and Additional file 1: Fig. S3). Correcting the time course of ^18^F-activity by the fractions of intact **[**^**18**^**F]7b** obtained by radio-HPLC or radio-TLC (Additional file 1: Fig. S3 inset) yielded the time courses for the activity concentration of **[**^**18**^**F]7b** in the blood, which was converted to the time course for the molar concentration of **[**^**18**^**F]7b** + **7b** (Fig. 3B). These time courses appeared to be biphasic, which is further supported by the respective semi-logarithmic plot in which two straight lines can be drawn (Fig. 3B inset). Analysis by linear regression provided values of 0.04 min^−1^ and 17.2 min for the elimination rate constant (*k*_el_) and the elimination half-life, respectively, based on the radio-HPLC data for the degradation of **[**^**18**^**F]7b**. In this context, comparable values for *k*_el_ were obtained based on the radio-TLC data (Additional file 1: Table S1). Furthermore, the volume of distribution (V_d_ = 1,383 mL/kg) and the plasma clearance (plasma CL = 56 mL/kg/min) were calculated (Additional file 1: Table S1). Analyzing the distribution of ^18^F-activity in the blood components at different time points revealed that ≈30% was bound to erythrocytes while ≈60% was located in the plasma fraction. This ratio appeared to be stable from 10 to 120 min *p.i.* Within the plasma fraction, almost all ^18^F-activity appeared to be in the supernatant and not protein-bound (Additional file 1: Fig. S4). At 20 min *p.i.* the fraction of unmodified **[**^**18**^**F]7b** was comparable in both blood components (> 40%), while ≥ 90% of **[**^**18**^**F]7b** remained intact when incubating the radiotracer in blood in vitro (Additional file 1: Fig. S5). Radio-HPLC chromatograms of urine and intestinal content 120 min *p.i.* revealed a variety of more hydrophilic metabolites and only marginal amounts of residual intact **[**^**18**^**F]7b** (8 and 7%, Fig. 3A). It is worth noting that the radio-HPLC chromatograms of blood samples (Fig. 3A) resemble that of the urine with regards to the retention time range. In contrast, a different radiometabolite profile was seen in the intestinal content. The time range for the elution of these radiometabolites was rather narrow and one major radiometabolite was observed.

**Fig. 4:**
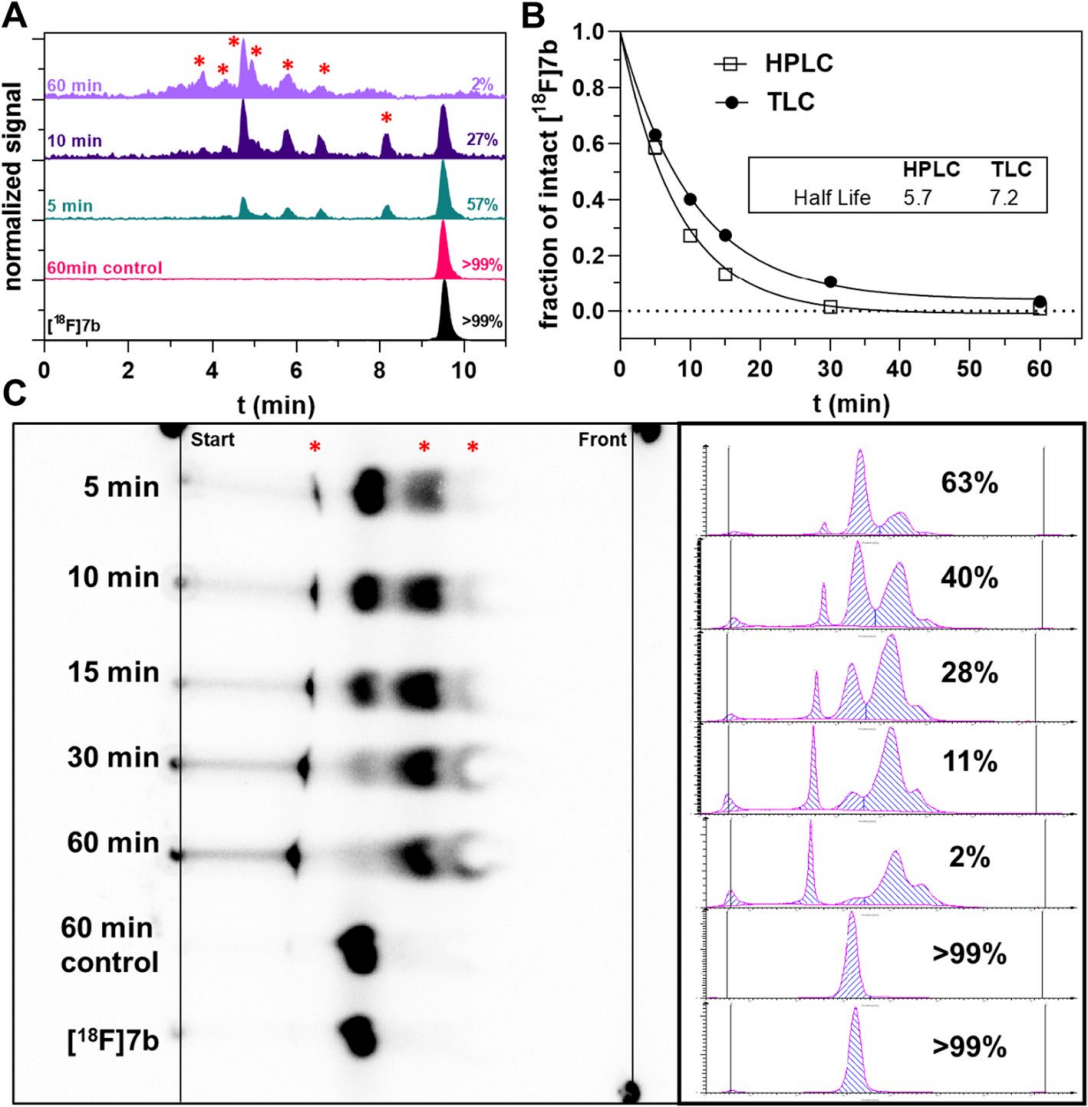
Metabolization of **[**^**18**^**F]7b** toward murine liver microsomes. **A**, **C** Exemplary radio-HPLC chromatograms (**A**) and radio-TLC (**C**) of **[**^**18**^**F]7b** after incubation with murine liver microsomes (MLMs) for different time periods. Radiometabolites are indicated by red asterisks.** B** Time course of residual intact [^18^F]7b toward incubation with MLMs determined by radio-HPLC and radio-TLC including nonlinear regression according to one-phase decay. The calculated half-life values are given in the box (in min). Conditions: 10 mM PBS (pH 7.4), 1 mg/mL MLM, 2 mM NADPH, 108 MBq/mL or 4.1 µM **[**^**18**^**F]7b**, 0.2% ethanol (v/v), for control conditions NADPH was replaced by PBS

To investigate the oxidative metabolism of **[**^**18**^**F]7b**, in vitro studies using mouse liver microsomes (MLM) were performed (Fig. 4). A variety of hydrophilic metabolites were formed upon incubation and a much better resolution of these radiometabolites was obtained with radio-HPLC analysis (7 radiometabolites) compared to radio-TLC analysis (3 radiometabolites and a spot of ^18^F-activity of increasing intensity over time at the baseline). However, the half-life of degradation calculated from the plot of residual intact **[**^**18**^**F]7b** obtained by radio-HPLC and radio-TLC was similar (5.7 and 7.2 min). Furthermore, the *K*_m_ and V_max_ values for the degradation of **[**^**18**^**F]7b** were estimated by following its time-dependent degradation at varying molar concentrations with a constant activity concentration. Replotting the initial rates (at t = 0 min) obtained from the time courses against the total concentration of **7b** and analysis by nonlinear regression according to the Michaelis–Menten equation yielded a *K*_m_ value of 1.5 µM and a V_max_ value of 0.37 µM/min (Additional file 1: Fig. S6). For both MLM studies, *i.e.* t_1/2_ and V_max_/*K*_m_ approach, the predicted intrinsic clearance values (pred. CL_int_) were calculated, which yielded values of 772 mL/kg/min and 1,610 mL/kg/min for the t_1/2_ and V_max_/*K*_m_ approach, respectively (Additional file 1: Table S2). Comparing the profiles of radiometabolites observed ex vivo and toward MLMs using the same HPLC system and method revealed that a significantly smaller range of radiometabolites is formed in the presence of MLMs under oxidative conditions (Additional file 1: Fig. S7). For characterizing the structures of radiometabolites, UPLC-MS/MS analyses were performed for carrier-added MLM incubations under oxidative conditions, after decay of ^18^F-activity. A total of eight potential metabolites has been detected (Additional file 1: Fig. S8). For four metabolites, the [M + H]^+^ signal is ≈16 amu higher than that of **7b**, which indicates their mono-hydroxylated status. Based on their fragment ion pattern compared to that of **7b**, two of these hydroxylated metabolites bear the hydroxy group at the pyridylpiperazine moiety, one likely at the benzyl moiety and one at the lysine side chain. For two metabolites, the [M + H]^+^ signal is reduced by ≈2 amu compared to **7b** with one being supposed to originate from hydroxy-defluorination and the other from dehydrogenation at the piperazine ring. Two metabolites could not be assigned to a potential structure. As a potential authentic metabolite formed by oxidative biotransformation, the respective pyridine-*N*-oxide of **7b**, **7b-*N*-oxide**, was synthesized by treatment of **7b** with *meta*-chloroperoxybenzoic acid in CH_2_Cl_2_ (Supporting Information). Based on comparing the HPLC retention times, **7b-*N*-oxide** (or **[**^**18**^**F**]**7b-*N*-oxide**) appeared to be a potential (radio)metabolite in vivo (Additional file 1: Fig. S9), but was most likely not formed in the MLM incubations (Additional file 1: Fig. S10). In addition, simultaneous treatment of **[**^**18**^**F]7b** with MLMs under oxidative and glucuronidation conditions showed the formation of at least one glucoronidated radiometabolite (Additional file 1: Fig. S11), for which glucuronidation occurred at the pyridylpiperazine moiety as seen by UPLC-MS/MS analysis (Additional file 1: Fig. S12). In contrast, **[**^**18**^**F]7b** without prior oxidation is not susceptible to glucuronidation (data not shown).

### *Cell uptake and release of [*^***18***^***F]7b***

Prior to selecting a suitable tumor model for evaluating the targetability of TGase 2 in vivo with **[**^**18**^**F]7b**, its cell uptake was characterized for different tumor cell lines over 60 min (Additional file 1: Fig. S13). For this purpose, four TGase 2-positive cell lines, *i.e.* A375, A375-hS100A4, NCI-H292, and MDA-MB-231, as well as one TGase 2-negative cell lines, *i.e.* MeWo, were selected based on recently published results from WesternBlot analysis and TGase 2 activity measurements in cell lysates (Hauser et al. 2022). Cell uptake was also studied in the presence of 10 µM **7b** or 100 µM verapamil. For all cell lines, a time-dependent increase of cell-bound ^18^F-activity was observed reaching values between 5 and 10%ID/mg after 60 min of incubation. Furthermore, the presence of an excess of **7b** led to a decrease of cell-bound activity for all cell lines, in particular at 30 and 60 min. The effect of verapamil was not uniform. For A375 and A375-hS100A4 the cell-bound activity was slightly increased, while for NCI-H292, MDA-MB-231 and MeWo, a diminished cell-bound activity was detectable in the presence of this agent. In addition to cell uptake, the release of **[**^**18**^**F]7b** from A375, A375-hS100A4 and MeWo cells after incubation for 60 min was characterized, again in the presence and absence of verapamil. A decrease in cell-bound activity with a concomitant increase in ^18^F-activity in the supernatant can be deviated, which seemed not to be affected by the presence of verapamil (Additional file 1: Fig. S14).

### Biodistribution and PET imaging in tumor bearing mice

For evaluating the suitability of **[**^**18**^**F]7b** for targeting of tumor-associated TGase 2, tumor xenograft models using A375-hS100A4 and MeWo cells were established. These two malignant melanoma cell lines are TGase 2-positive and TGase 2-negative, respectively. Similar to the in vivo characterization of **[**^**18**^**F]7b** in healthy mice, the distribution of **[**^**18**^**F]7b** in the tumor bearing mice was analyzed by ex vivo biodistribution and PET imaging studies. As expected, the overall biodistribution resembles the results for the healthy mice. In fact, an increase in retained ^18^F-activity from 5 to 60 min *p.i.* is seen for the bone tissue (Additional file 1: Fig. S15). The uptake in both tumor types is higher at 5 min compared to 60 min *p.i.* and tumor/muscle ratio of 1.4 and 1.0 can be calculated for A375-hS100A4 and MeWo, respectively. The results from PET imaging support the obtained data for the ex vivo biodistribution. In particular, a continuous decrease of ^18^F-activity in the tumor tissue was observed. However, again a slightly higher tumor/muscle ratio can be derived for the A375-hS100A4 tumor compared to the MeWo tumor (Fig. 3). Additionally, uptake in both tumor models was studied by ex vivo biodistribution in the presence of non-radioactive **7b** (A_m_ = 0.3 GBq/µmol for c.a. level compared to 52 GBq/µmol for n.c.a. level) at 60 min p.i., which revealed a comparable tumor uptake under n.c.a. and c.a. levels (Additional file 1: Fig. S15).

## Discussion

TGase 2 represents an emerging theranostic target for different pathophysiological processes with irreversible inhibitors directed toward the transamidase activity being the primary focus as targeting molecules. In this context, the peptidomimetic inhibitor ZED1227, which is equipped with an α,β-unsaturated methyl ester as Michael acceptor for the active site cysteine residue of TGase 2 (Büchold et al. 2022), is in clinical evaluation for the treatment of celiac disease. The results of a recently published randomized trial showed that the application of ZED1227 for treatment of patients with celiac disease is well-tolerated and led to an attenuated damage of the duodenal mucosa (Schuppan et al. 2021). These results might also stimulate the development of theranostic agents for other pathophysiological processes involving TGase 2. While the molecular function of TGase 2 in celiac disease is well-understood, its detailed role for tumor development and progression appears to be complex. Therefore, imaging probes that allow for the in vivo detection of tumor-associated TGase 2 and in particular via its transamidase activity are in demand.

Herein, results for the preclinical characterization of the *N*^ε^-acryloyllysine piperazide **[**^**18**^**F]7b** are reported. Compound **[**^**18**^**F]7b** is a potent and selective irreversible inhibitor of TGase 2 whose capability of targeting TGase 2 at the cellular level has recently been demonstrated by us (Wodtke et al. 2021). Although a reliable radiosynthesis under base-free conditions using the “minimalist approach” of Richarz et al*.* (2014) was established for **[**^**18**^**F]7b**, we sought for an alternative access to **[**^**18**^**F]7b** which requires less amount of precursor. This was realized by taking advantage of our previously reported microliter scale radiofluorination approach (Laube et al. 2021), but required the radiofluorination under basic conditions (K_2_CO_3_ as base). As expected, the formation of radiolabeled side-products was more pronounced compared to the base-free approach (Additional file 1: Fig. S1) due to the known base sensitivity of the phenylacetyl moiety (Wodtke et al. 2021). This in turn might account for the slightly lower radiochemical yield (17 ± 7% *versus* 34 ± 14%, Scheme 1). Considering the elaborative synthesis of the respective trimethylammonio precursor **8**, the new radiosynthesis under basic conditions in a low reaction volume and thus low precursor demand appears more convenient compared to our previously reported radiosynthesis under base-free conditions.

Although TGase 2 is ubiquitously expressed, in particular in liver and kidneys (Wodtke et al. 2021), the data from ex vivo biodistribution and PET imaging studies in healthy mice did not indicate an undesired permanent ^18^F-activity enrichment in those organs (Figs. 1 and 2). This supports the general view that the transamidase activity of TGase 2 is usually latent under physiological conditions. The initially high uptake in the liver and the high percentage of ^18^F-activity in the intestinal content suggest that the elimination of **[**^**8**^**F]7b** occurs predominantly via the hepatobiliary excretion route. Considering the moderate lipophilicity of **[**^**18**^**F]7b** (logD_7.4_ of 2.1) (Wodtke et al. 2021), this pharmacokinetic behavior was expected. However, in contrast to most of the organs, there was a clear accumulation of ^18^F-activity in the bone tissue. Even though TGase 2-mediated protein crosslinking has been proposed to be crucial for bone mineralization (Heath et al. 2001; Kaartinen et al. 2002), activity retention in the bone for ^18^F-labeled tracers is a strong indication toward their metabolic defluorination. This is supported by the MLM studies, in which an increasing amount of [^18^F]F^−^ was noted by radio-TLC analyses (Fig. 4) (Laube et al. 2020). Moreover, UPLC-MS/MS analysis indicated the formation of a hydroxy-defluorinated metabolite (Additional file 1: Fig. S8). In vivo, the ^18^F-activity enrichment in the bone tissue could be blocked by pretreatment with ketoconazole, which further supports the assumption of metabolically released [^18^F]fluoride and indicates the involvement of CYP3A enzymes for this metabolic transformation (Figs. 1 and 2). While C–F bonds are usually more metabolically stable than C–H bonds, in particular in case of aromatic moieties (Shah and Westwell 2007), there are accumulating examples for defluorinations from different chemical entities (Guengerich 2001; Pan 2019). A prominent example is the hydroxy-dehalogenation of 4-haloanilines, which is fastest under substrate saturation for 4-fluoroaniline (Cnubben et al. 1995). Furthermore, the norepinephrine transporter tracer [^18^F]NS12137, which bears a 6-fluoro-2-pyridinyloxy residue, underlies defluorination (Kirjavainen et al. 2018). Therefore, it can be postulated that 2-fluoropyridyl moieties with electron-donating substituent in 6-position are generally prone to metabolic defluorination. In line with this hypothesis, the serotonin receptor ligand [^18^F]6FPWAY, whose pyridine ring bears an identical atomic substitution pattern to **[**^**18**^**F]7b** with the striking difference that the electron donating capability of the nitrogen atom is attenuated by acylation, shows no sign of rapid defluorination (McCarron et al. 2004). Regarding the involvement of CYP enzymes for defluorination from aromatic systems, sunitinib was recently shown to be oxidatively defluorinated by CYP1A2 and CYP3A4 (Amaya et al. 2018; Burnham et al. 2022) and detailed investigations toward potential mechanism of this transformation were carried out at high level of theory (Zhang et al. 2023). A potential mechanism for the observed hydroxy-defluorination of **7b** is formulated in the Supporting Information (Additional file 1: Fig. S16) (Meunier et al. 2004; Denisov et al. 2005). It is worth noting that the pretreatment with ketoconazole not only diminished the ^18^F-activity uptake in the bone tissue but led also to a significant enrichment of ^18^F-activity in most of the other organs and reduced activity in the urine and intestinal content (Figs. 1 and 2). This might result from the inhibitory activity of ketoconazole toward the transmembrane transporter P-glycoprotein (Kim et al. 1999), which is expressed in a variety of organs including liver, intestine, brain and kidney (Thiebaut et al. 1987; Staud et al. 2010), preventing the active export of **[**^**18**^**F]7b** and/or its metabolites from these tissues. In this context, Wityak et al*.* (2012) recently attributed the analogous compound bearing a methyl instead of a fluorine atom in position 6 of the pyridine ring to underly a high active efflux mediated by the P-glycoprotein. Therefore, the in vivo behavior of **[**^**18**^**F]7b** in the presence of ketoconazole is affected by diminished metabolism and excretion resulting from the inhibition of CYP3A enzymes and P-glycoprotein, respectively.

Apart from defluorination, **[**^**18**^**F]7b** underlies metabolic transformations to various radiometabolites as seen from analyzing the urine and the intestinal content as well as from MLM studies (Figs. 3A and 4). Previously, we investigated the stability of **7b** toward MLM and noted that mono-hydroxylation occurs at multiple sites of the molecule (Wodtke et al. 2018), which was confirmed herein (Additional file 1: Fig. S8). Regarding hydroxylation at the pyridylpiperazine moiety, we speculated that pyridine-*N*-oxygenation, which is a prominent metabolization of pyridine in vivo (Damani et al. 1982), could have occurred for **[**^**18**^**F]7b**. Indeed, this respective radiometabolite could have been formed in vivo, but not toward MLMs (Additional file 1: Figs. S8 and S9). Moreover, MLM incubations under oxidative/glucuronidation conditions revealed that also phase II transformations occur after prior oxidation (Additional file 1: Figs. S11 and S12). The larger profile of radiometabolites observed in the intestinal content and urine compared to the MLM incubations suggests that **[**^**18**^**F]7b** is subject to further metabolic transformations in vivo. In this context, methylation, in particular at the pyridine ring, is likely to occur as also known for pyridine (Damani et al. 1982). Moreover, there are various biotransformation and bioactivation reactions known for piperazine rings, including *N*-dealkylation, *N*-oxidation, lactam formation and ring cleavage and opening (Masic 2011; Bolleddula et al. 2014). The estimation of the *K*_m_ value for the degradation of **[**^**18**^**F]7b** by substrate depletion as described by Obach and Reed-Hagen for non-radioactive compounds (Obach and Reed-Hagen 2002) yielded a remarkably low Michaelis constant in the single-digit µM range (1.5 µM, Additional file 1: Fig. S6). Based on the experimental results for the MLM incubations, predicted intrinsic clearance values (pred. CL_int_) were calculated. Considering the concentration of **[**^**18**^**F]7b** for the half-life approach (3–4 µM, Fig. 4) and the apparent *K*_m_ value of 1.5 µM, the necessary assumption for predicting the intrinsic clearance value, i.e. [S] <  < *K*_m_ (Obach et al. 1997), is actually not fulfilled and the V_max_/*K*_m_ approach might provide thus a more reliably estimation for the intrinsic clearance. A value of 1,610 mL/kg/min for the intrinsic clearance in mice were obtained. In line with this apparently high intrinsic clearance in mice, a medium to high value for the plasma clearance (56 mL/kg/min) (Toutain and Bousquet-Melou 2004) in a Wistar rat was determined based on the blood sampling data. In this context, we should mention that the derivation of pharmacokinetic parameters from blood sampling data of one animal, herein a rat, does certainly not provide completely reliable data, but might rather serve as an initial estimation.

For the selection of a suitable tumor model to study the targeting of tumor-associated TGase 2 in vivo, the uptake of **[**^**18**^**F]7b** in different tumor cell lines was initially characterized. A clear TGase 2-mediated cell uptake was not detectable, in particular as the actual TGase 2-negative cell line MeWo showed a comparable time-dependent cell uptake as the TGase 2-positive cell lines A375, A375-hS100A4, NCI-H292, and MDA-MB-231 (Additional file 1: Fig. S13). Furthermore, the presence of non-radioactive **7b** did not lead to significant attenuation of radiotracer binding. To potentially increase the intracellular concentration of **[**^**18**^**F]7b** and thus favor a reaction with TGase 2, the cell uptake was also studied in the presence of verapamil, which is known to bind to P-glycoprotein (Yusa and Tsuruo 1989) and could thus interfere with P-gp-mediated efflux of **[**^**18**^**F]7b**. However, the presence of verapamil had apparently no significant effect on the **[**^**18**^**F]7b** uptake. The apparent absence of a TGase 2-mediated cell uptake is in line with our previous radio-SDS-PAGE experiments, in which no radiotracer-TGase -complex could be detected after treatment of intact A375-hS100A4 cells with **[**^**18**^**F]7b** for up to 4 h (Wodtke et al. 2021). Despite the missing correlation between TGase 2 activity and cell binding of **[**^**18**^**F]7b**, we hypothesized that the activity status of tumor-associated TGase 2 in vivo might be altered and is ideally higher to that in cell monolayers, considering in particular the formation of a tumor microenvironment at the tissue level (Lee et al. 2015). Therefore, we continued with the preclinical evaluation in tumor-bearing mice.

**Fig. 5:**
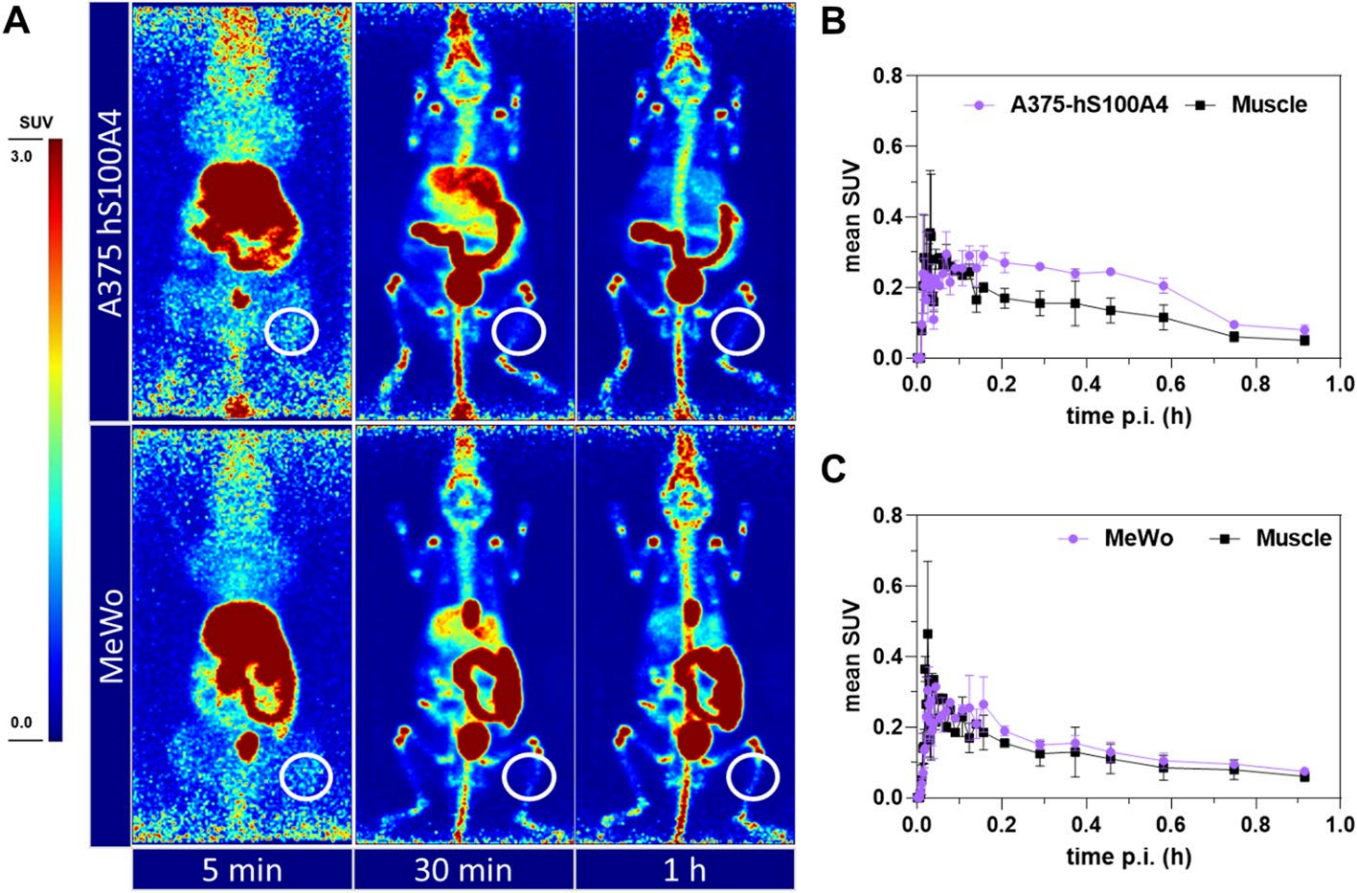
PET imaging for **[**^**18**^**F]7b** in tumor-bearing mice. **A** PET images at selected time points after intravenous injection of **[**^**18**^**F]7b** in mice bearing A375-hS100A4 (upper panel, 8.91 MBq) or MeWo (lower panel, 10.04 MBq) tumors on the right leg. Images are presented as maximum intensity projections and shown at a common scale (SUV 0–3). Indicated time points correspond to the following time frames: 5 min [4–6 min], 30 min [25–30 min], and 1 h [50–60 min]. Anatomical positions of the tumors used for SUV quantification are shown as white circles. Of note, the SUV scale for the images was set from 0.0 to 3.0 in contrast to the scale used for the PET images in Fig. 2A (0.0–8.0). **B**, **C** Standard uptake values (SUV, decay-corrected) for A375-hS100A4 (**B**) and MeWo tumors (**C**) with the respective muscle tissue as reference as a function of time (up to 1 h). Data shown are mean values of two mice each, which received a single injection of **[**^**18**^**F]7b**

For the in vivo evaluation, melanoma tumor xenograft models based on A375-hS100A4 (TGase 2 +) and MeWo (TGase 2 −) cells were chosen. For tumor xenografts derived from wild-type A375 and MeWo cells, we previously confirmed the presence and absence of TGase 2 by immunohistochemical staining. Furthermore, a strong TGase 2-mediated binding of **[**^**18**^**F]7b** to A375 tumor sections was detectable by autoradiography while binding to MeWo tumor sections was negligible (Wodtke et al. 2021). For A375-hS100A4 cells, the TGase 2 level was checked by WesternBlot analysis and the amount of reactive TGase 2 was assessed by binding of **[**^**18**^**F]7b** to TGase 2 in cell lysates and living cells using radio-SDS-PAGE (Wodtke et al. 2021). These data indicated that the transfection of the wild-type A375 cells did not affect the synthesis and activity status of TGase 2. In this context, S100A4 is another Ca^2+^-binding protein of significant importance in metastasizing tumors and is generally considered as biomarker for the tumor progression (Ismail et al. 2008, 2010; Bresnick et al. 2015). In previous studies, we showed that A375 cells stably transfected with human S100A4 (i.e. A375-hS100A4) actively secrete this protein in the extracellular space and both its synthesis and secretion resulted in prometastatic activation of A375 cells (Herwig et al. 2016a, b; Herwig et al. 2016a, b). Moreover, S100A4 was reported to be a substrate of TGase 2 and the TGase 2-catalyzed cross-linking of S100A4 had a positive impact on the migration behavior of mammary tumor cells (Wang and Griffin 2013; Biri et al. 2016). Therefore, a A374-hS100A4-derived tumor model appeared more appropriate than a model based on wild-type A375 cells to detect tumor-associated TGase 2. Ex vivo biodistribution and PET imaging showed a comparable uptake of ^18^F-activity in both tumors, which was rapidly declining over time (Fig. 5 and Additional file 1: Fig. S15). This indicates that no irreversible trapping, as expected upon irreversible binding to TGase 2, and thus, no successful TGase 2 targeting has occurred. In accordance to this, blocking of the observed tumor uptake with non-radioactive **7b** was not successful for both tumor models. Considering the cell uptake studies, one reason for the absent TGase 2 targeting might be the limited concentration of transamidase-active TGase 2 at the tumor site. However, the actual targeting might be also affected by the rapid distribution and elimination of **[**^**18**^**F]7b** including its pronounced metabolization, which lowers the available concentration of reactive radiotracer in the blood circulation. In this context, a largely constant portion of around 30% of ^18^F-activity in the blood was found to be located in the erythrocytes (Additional file 1: Fig. S4), which is generally not uncommon for drugs (Hinderling 1997), but might further contribute to a low available plasma concentration of **[**^**18**^**F]7b**. In this context, non-specific reaction with SH groups of plasma proteins can be largely excluded as the binding to plasma proteins is quite low (Additional file 1: Fig. S4). This is in accordance to the low GSH reactivity of **[**^**18**^**F]7b** in vitro (Wodtke et al. 2021).

There are only two other radiolabeled inhibitors of TGase 2 that underwent preclinical evaluation in vivo (Scheme S1 in Supporting Information) (van der Wildt et al. 2018). Compound **[**^**11**^**C]1** belongs to the same chemotype of *N*^ε^-acryloyllysine piperazides as **[**^**18**^**F]7b** with a methyl group at the position of the fluorine atom. Results from ex vivo biodistribution in mice and rats, PET imaging and ex vivo metabolite analysis appear to be similar to the data obtained for **[**^**18**^**F]7b** herein. In particular, a dominant hepatobiliary excretion and pronounced metabolization was seen (24% and 29% intact tracer 45 min *p.i.* in mouse and rat plasma ex vivo, respectively) (van der Wildt et al. 2016, 2018). In contrast, **[**^**18**^**F]2** is a peptidic ^18^F-labeled TGase 2 inhibitor and shows a higher uptake in kidneys than in liver. **[**^**18**^**F]2** is also rapidly metabolized in vivo but to exclusively one radiometabolite (98% after 15 min), which was identified to originate from hydrolysis of the methyl ester functionality **[**^**18**^**F]2**. This radiometabolite was also shown to be still a potent inhibitor of TGase 2 (van der Wildt et al. 2017a, b; van der Wildt et al. 2018). In mice bearing MDA-MB-231 tumors, the tumor uptake curve for **[**^**11**^**C]1** resembles that of **[**^**18**^**F]7b**, while the uptake of **[**^**18**^**F]2** increased over time. Furthermore, blocking of **[**^**18**^**F]2** uptake with non-radioactive **2** and another chemotype of TGase 2-inhibitor (ERW1041E) was successful, which suggests that the tumor uptake was indeed mediated by TGase 2. Although data for the arterial blood clearance of **[**^**18**^**F]2** were not provided, it is reasonable to assume that for **[**^**18**^**F]2** the decline in concentration of TGase 2-reactive radiotracer (**[**^**18**^**F]2** and its radiometabolite) is slower compared to **[**^**11**^**C]1** or **[**^**18**^**F]7b**. Moreover, the inhibitory potency by means of the inactivation constant *k*_inact_/*K*_I_ might be at least by a factor of 10 higher for **2** compared to **1** and **7b** as shown for other peptidic inhibitors bearing a diazomethyl ketone warhead (Hausch et al. 2003; Pinkas et al. 2007; Wodtke et al. 2020). Together, higher metabolic stability and more rapid target binding should favor the targeting of tumor-associated TGase 2.

Apart from radiolabeled inhibitors, Ackermann et al*.* (Ackermann et al. 2023) recently reported on the ^18^F-labeling of the glutamine donor peptide T26, which was shown to be largely selective for crosslinking by TGase 2 (Sugimura et al. 2006; Hitomi et al. 2009). This 13mer peptide with the sequence H-HQSYVDPWMLDH-OH was C-terminally elongated by an 5-azidopentanoyl linker and radiolabeling was achieved by reaction with [^18^F]FBz-DBCO as prosthetic group via copper-free click reaction. Evaluation of the resulting [^18^F]FBz-DBCO-peptide in mice bearing MDA-MB-231 (TGase 2 +) or MCF-7 (TGase 2 −) tumors revealed that a radiotracer accumulation at the tumor site was not observed for both models, which would have been expected to occur upon TGase 2-catalzyed incorporation of the radiotracer into cellular proteins. However, the tumor washout over 90 min *p.i.* was slower for MDA-MB-231 compared to MCF-7, but unequivocal evidence for TGase 2-mediated uptake could not be obtained in this study.

## Conclusion

Compound **[**^**18**^**F]7b**, an ^18^F-labeled irreversible inhibitor of TGase 2, was recently shown to be a valuable radiometric tool for studying mechanistic aspects of recombinant TGase 2 and for targeting of TGase 2 in biological matrices such as cell lysates and tissue sections. The herein presented radiopharmacological characterization revealed that its use for targeting of tumor-associated TGase 2 is limited due to a rapid distribution in and elimination from the organism including a pronounced metabolization. From a radiochemical perspective, this requires further structural modifications of **[**^**18**^**F]7b**. In addition, radionuclides of longer half-life than that of carbon-11 or fluorine-18 should be considered for irreversible inhibitors as the assessment of a potentially low tumor uptake becomes easier at later time points *p.i.* when washout from normal tissue is more progressed. For this purpose, we recently developed ^123^I-labeled inhibitors, which are currently subject of preclinical investigations (Donat et al. 2022).

### Supplementary Information

**Additional file 1**. **Figure S1**: Exemplary radio-HPLC chromatogram for the purification of **[**^**18**^**F]7b**; **Figure S2**: Exemplary UV- and radio-HPLC as well as radio-TLC chromatograms of finally formulated **[**^**18**^**F]7b**; **Figure S3**: Time course of residual ^18^F-activity and fraction of **[**^**18**^**F]7b** in blood ex vivo; **Figure S4**: Ex vivo distribution of ^18^F-activity in blood components of a Wistar rat; **Figure** **S5**: Stability of **[**^**18**^**F]7b** in plasma and erythrocytes after incubation in vitro and in vivo; **Figure S6**: *K*_m_ and *V*_max_ determination for the degradation of **[**^**18**^**F]7b** by murine liver microsomes; **Figure S7**: Comparison of the profiles of radiometabolites ex vivo and toward MLMs; **Figure S8**: UPLC-MS/MS analysis of carrier added MLM incubations under oxidative conditions; **Figure S9**: Comparison of HPLC retention times for **7b-N-oxide** and the profile of radiometabolites observed in vivo; **Figure S10**: Comparison of HPLC retention times for **7b-*N*-oxide** and the profile of radiometabolites observed toward MLM; **Figure S11**: Treatment of **[**^**18**^**F]7b** with MLMs under oxidative conditions in the presence of Alamethicin and UDPGA; **Figure S12**: UPLC-MS/MS analysis of carrier added MLM incubations under oxidative/glucoronidation conditions; **Figure S13**: Uptake of **[**^**18**^**F]7b** in different tumor cell lines; **Figure S14:** Release of **[**^**18**^**F]7b** from different tumor cell lines; **Figure S15**: Biodistribution of **[**^**18**^**F]7b** in tumor-bearing mice; **Figure S16**: Hypothetical mechanism of the observed CYP-mediated ^18^F-defluorination of **[**^**18**^**F]7b** assuming plausible hydroxy-defluorination; **Scheme** **S1**: Structures of previously reported radiotracer for TGase 2; **Table S1**: Summary of pharmacokinetic parameters for **[**^**18**^**F]7b** determined in a healthy Wistar rat; **Table S2**: Summary of pharmacokinetic parameters for **[**^**18**^**F]7b** derived from experiments with murine liver microsomes.

## Data Availability

All data generated or analyzed during this study are included in this published article and its Additional files.

## Abbreviations

%ID: Percentage of injected dose
ABP: Activity-based probe
i.v.: Intravenously
GTP-γS: Guanosine-5′-(γ-thio)-triphosphate
MLM: Murine liver microsomes
P-gp: P-glycoprotein
SUV: Standardized uptake value
TCI: Targeted covalent inhibitor
TGase2: Transglutaminase 2 (tissue transglutaminase)
