# Supplementary material for: Preclinical evaluation of an 18F-labeled Nε-acryloyllysine piperazide for covalent targeting of transglutaminase 2
Source: EJNMMI Radiopharm Chem. 2024 Jan 2;9:1. doi: 10.1186/s41181-023-00231-1 (PMC10761660; doi:10.1186/s41181-023-00231-1)
Supplement: Supplementary file 1 — Additional file 1. Figure S1: Exemplary radio-HPLC chromatogram for the purification of [18F]7b; Figure S2: Exemplary UV- and radio-HPLC as well as radio-TLC chromatograms of finally formulated [18F]7b; Figure S3: Time course of residual 18F-activity and fraction of [18F]7b in blood ex vivo; Figure S4: Ex vivo distribution of 18F-activity in blood components of a Wistar rat; Figure S5: Stability of [18F]7b in plasma and erythrocytes after incubation in vitro and in vivo; Figure S6: Km and Vmax determination for the degradation of [18F]7b by murine liver microsomes; Figure S7: Comparison of the profiles of radiometabolites ex vivo and toward MLMs; Figure S8: UPLC-MS/MS analysis of carrier added MLM incubations under oxidative conditions; Figure S9: Comparison of HPLC retention times for 7b-N-oxide and the profile of radiometabolites observed in vivo; Figure S10: Comparison of HPLC retention times for 7b-N-oxide and the profile of radiometabolites observed toward MLM; Figure S11: Treatment of [18F]7b with MLMs under oxidative conditions in the presence of Alamethicin and UDPGA; Figure S12: UPLC-MS/MS analysis of carrier added MLM incubations under oxidative/glucoronidation conditions; Figure S13: Uptake of [18F]7b in different tumor cell lines; Figure S14: Release of [18F]7b from different tumor cell lines; Figure S15: Biodistribution of [18F]7b in tumor-bearing mice; Figure S16: Hypothetical mechanism of the observed CYP-mediated 18F-defluorination of [18F]7b assuming plausible hydroxy-defluorination; Scheme S1: Structures of previously reported radiotracer for TGase 2; Table S1: Summary of pharmacokinetic parameters for [18F]7b determined in a healthy Wistar rat; Table S2: Summary of pharmacokinetic parameters for [18F]7b derived from experiments with murine liver microsomes. [file 41181_2023_231_MOESM1_ESM.docx]

**Supporting Information**

**Preclinical evaluation of an ^18^F-labeled N^ε^-acryloyllysine piperazide for covalent targeting of transglutaminase 2**

Robert Wodtke^a*^, Markus Laube^a^, Sandra Hauser^a^, Sebastian Meister^a^,
Friedrich-Alexander Ludwig^b^, Steffen Fischer^b^, Klaus Kopka^a,b,c^,
Jens Pietzsch^a,c^, Reik Löser^a,c*^

*[a] Helmholtz-Zentrum Dresden-Rossendorf, Institute of Radiopharmaceutical Cancer Research, Bautzner Landstraße 400, 01328 Dresden, Germany*

*[b] Helmholtz-Zentrum Dresden-Rossendorf, Institute of Radiopharmaceutical Cancer Research, Permoserstraße 15, 04318 Leipzig, Germany*

*[c] Technische Universität Dresden, School of Science, Faculty of Chemistry and Food Chemistry, Mommsenstraße 4, 01069 Dresden, Germany*

*Corresponding authors:

Robert Wodtke*,* [r.wodtke@hzdr.de](mailto:r.wodtke@hzdr.de) , Helmholtz-Zentrum Dresden-Rossendorf, Institute of Radiopharmaceutical Cancer Research, Bautzner Landstraße 400, 01328 Dresden, Germany;

Reik Löser, [r.loeser@hzdr.de](mailto:r.loeser@hzdr.de) *,* Helmholtz-Zentrum Dresden-Rossendorf, Institute of Radiopharmaceutical Cancer Research, Bautzner Landstraße 400, 01328 Dresden, Germany

Table of Contents

[Chemistry 3](#_Toc149504048)

[Synthesis and Characterization of **7b-N-oxide** 3](#_Toc149504049)

[Figure S1: Exemplary radio-HPLC chromatogram for the purification of [^18^F]7b 5](#_Toc149504050)

[Figure S2: Exemplary UV- and radio-HPLC as well as radio-TLC chromatograms of finally formulated [^18^F]7b 6](#_Toc149504051)

[UV-HPLC chromatogram (λ=254 nm) 6](#_Toc149504052)

[Radio-HPLC chromatogram 6](#_Toc149504053)

[UV-HPLC chromatogram after addition of **7b** (“spiked”, λ=254 nm) 7](#_Toc149504054)

[Radio-TLC chromatogram 8](#_Toc149504055)

[Figure S3: Time course of residual ^18^F-activity and fraction of [^18^F]7b in blood ex vivo 9](#_Toc149504056)

[Figure S4: Ex vivo distribution of ^18^F-activity in blood components of a Wistar rat 10](#_Toc149504057)

[Figure S5: Stability of [^18^F]7b in plasma and erythrocytes after incubation in vitro and in vivo 11](#_Toc149504058)

[Figure S6: K_m_ and V_max_ determination for the degradation of [^18^F]7b by murine liver microsomes 12](#_Toc149504059)

[Figure S7: Comparison of the profiles of radiometabolites ex vivo and toward MLMs 13](#_Toc149504060)

[Figure S8: UPLC-MS/MS analysis of carrier added MLM incubations under oxidative conditions 14](#_Toc149504061)

[Figure S9: Comparison of HPLC retention times for 7b-N-oxide and the profile of radiometabolites observed in vivo 23](#_Toc149504062)

[Figure S10: Comparison of HPLC retention times for 7b-N-oxide and the profile of radiometabolites observed toward MLM 24](#_Toc149504063)

[Figure S11: Treatment of [^18^F]7b with MLMs under oxidative condition in the presence of Alamethicin and UDPGA 25](#_Toc149504064)

[Figure S12: UPLC-MS/MS analysis of carrier added MLM incubations under oxidative/glucoronidation conditions 26](#_Toc149504065)

[Figure S13: Uptake of [^18^F]7b in different tumor cell lines 28](#_Toc149504066)

[Figure S14: Release of [^18^F]7b from different tumor cell lines 29](#_Toc149504067)

[Figure S15: Biodistribution of [^18^F]7b in tumor-bearing mice 30](#_Toc149504068)

[Figure S16: Hypothetical mechanism of the observed CYP-mediated ^18^F-defluorination of [^18^F]7b assuming plausible hydroxy-defluorination. 32](#_Toc149504069)

[Scheme S1: Structures of previously reported radiotracers for TGase 2 33](#_Toc149504070)

[Table S1: Summary of pharmacokinetic parameters for [^18^F]7b determined in a healthy Wistar rat 34](#_Toc149504071)

[Table S2: Summary of pharmacokinetic parameters for [^18^F]7b derived from experiments with murine liver microsomes 35](#_Toc149504072)

[References for Supporting Information 36](#_Toc149504073)

Chemistry

Synthesis and Characterization of **7b-N-oxide**

To a solution of compound **7b** (5 mg, 10.4 µmol) in CH_2_Cl_2_ (500 µL) was added *meta*-chloroperbenzoic acid (77%, mixture with *meta*-chlorobenzoic acid; 2.56 mg; 11.4 µmole) and stirred at room temperature overnight. After 23 h, analysis of an aliquote by LC-MS has revealed that approximately 1/3 of **7b** was unconverted, for which the identical portion of oxidant was added again. After stirring for additional 4 h, only minor amounts of **7b** were detectable. The solvent was evaporated and the obtained residue was subjected to purification by semi-preparative HPLC using the identical system as in the radiosynthesis of **[^18^F]7b** equipped with a Jupiter Proteo C18 4 µm column (Phenomenex, 250×21.2 mm) as stationary phase. A binary gradient system of 0.1% trifluoroacetic acid in water (solvent A) and 0.1% trifluoroacetic acid in CH_3_CN (solvent B) served as the eluent. Gradient elution was performed using 25% eluent B for 5 min, 25% to 75% eluent B in 20 min, 75% to 95% eluent B in 1 min, 95% eluent B for 5 min, 95% to 25% eluent B in 1 min, and 25% eluent B for 10 min (total time of 42 min) at a flow rate of 10 mL/min. The product-containing fractions were combined, concentrated and lyophilized to obtain **7b-*N*-oxide** (trifluoroacetate salt; 1.1 mg, 17%) as white solid.

^1^H NMR (400 MHz, DMSO-*d*_6_) δ = 1.21 – 1.30 (m, 2H, C_γ_H_2_), 1.40-1.44 (m, 2H, C_δ_H_2_), 1.49-1.63 (m, 1H, C_β_*H*H), 1.65-1.72 (m, 1H, C_β_H*H*), 3.10 (q, *J* = 6.6 Hz, 2H, C_ε_H_2_), 3.39 – 3.53 (m, 3H, PhCH_2_, 1xpiperazine-H), 3.80 (t, *J* = 13.3 Hz, 1H, 1xpiperazine-H), 3.88 – 4.06 (m, 3H, 3xpiperazine-H), 4.11 – 4.36 (m, 2H, 2xpiperazine-H), 4.44 – 4.57 (m, 1H, 1xpiperazine-H), 4.72 (s, 1H, C_α_H), 5.50-5.61 (m, 1H, *H*HC=CH), 5.98-6.11 (m, 1H, H*H*C=CH), 6.13-6.24 (m, 1H, HHC=C*H*), 7.14 (d, *J* = 6.7 Hz, 1H, N_ε_H), 7.09 – 7.33 (m, 5H, Ph-H), 7.64 (dd, ^3^*J*_H,H_ = 8.2, ^3^*J*_H,F_ = 2.3 Hz, 1H, H-3 of pyridine), 8.06 (br s, 1H, N_ε_H), 8.14 (t, ^3^*J*_H,H_ ≈ ^3^*J*_H,F_ = 10.0 Hz, 1H, H-5 of pyridine), 8.39 – 8.56 (m, 2H, H-4 of pyridine, N_α_H).

^13^C NMR (101 MHz, DMSO-*d*_6_) δ = 22.57 (C_γ_), 28.80 (C_δ_), 31.35 (C_β_), 35.96 (2xpiperazine-CH2), 38.27 (C_ε_), 41.95 (PhCH_2_), 44.11, 44.25, 44.56, 48.13 (C_α_), 113.87 (broad, C-3, C-5 of pyridine), 124.76 (H_2_*C*=CH), 126.30 (C-4 of phenyl), 128.13, 128.96 (C-2, C-3 of phenyl), 131.85 (H_2_*C*=*C*H), 136.37 (C-1 of phenyl), 147.17 (broad, C-4 of pyridine), 157.65 (^3^*J*_C,F_ = 15.9 Hz, C-2 of pyridine), 164.42 (CONε), 164.58 (^1^*J*_C,F_ = 294.9 Hz, C-6 of pyridine), 169.77, 170.02 (CON). MS (ESI^+^): m/z calculated for C_26_H_33_FN_5_O_4_: 498.25 [M+H]^+^, found: 497.9, m/z calculated for C_26_H_32_FN_5_O_4_: 481.25 [M+H-OH^•^]^+^, found: 481.2.

Figure S1: Exemplary radio-HPLC chromatogram for the purification of [^18^F]7b


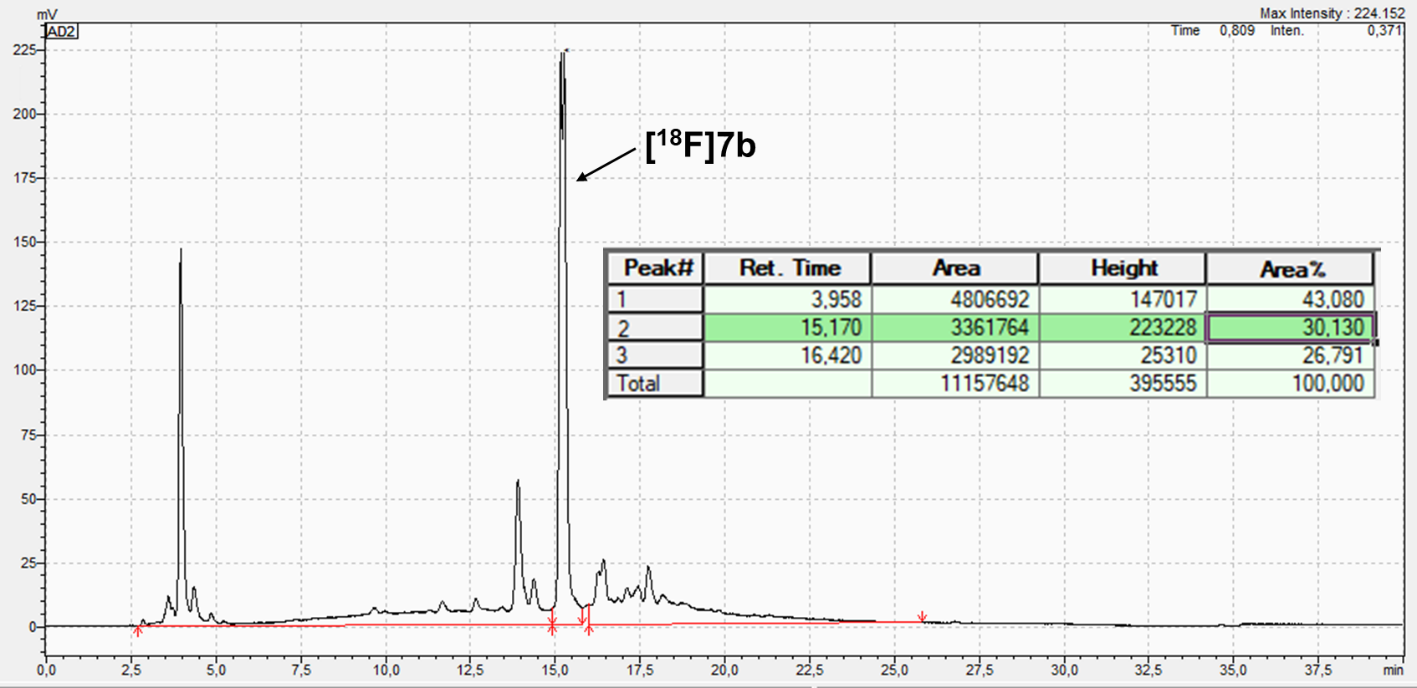


Figure S2: Exemplary UV- and radio-HPLC as well as radio-TLC chromatograms of finally formulated [^18^F]7b

UV-HPLC chromatogram (λ=254 nm)


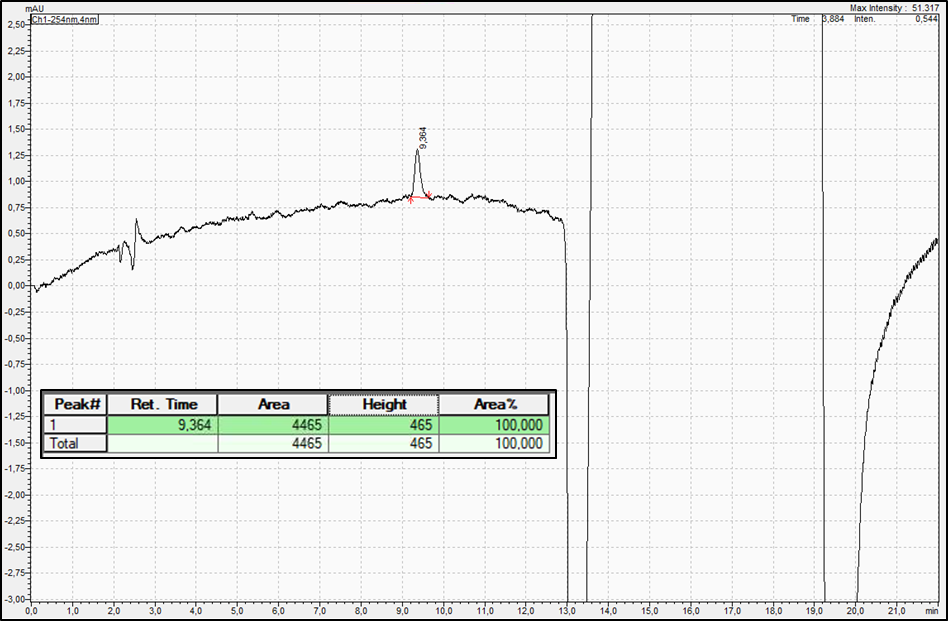


Radio-HPLC chromatogram


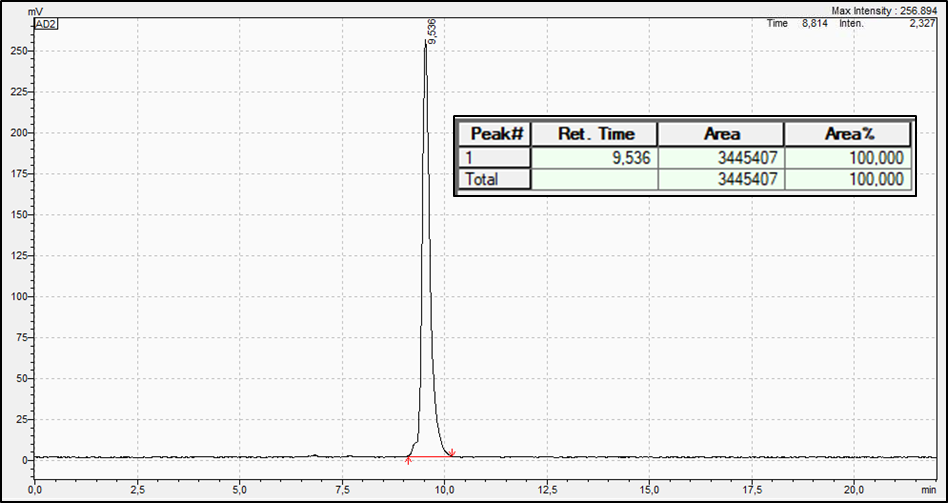


UV-HPLC chromatogram after addition of **7b** (“spiked”, λ=254 nm)


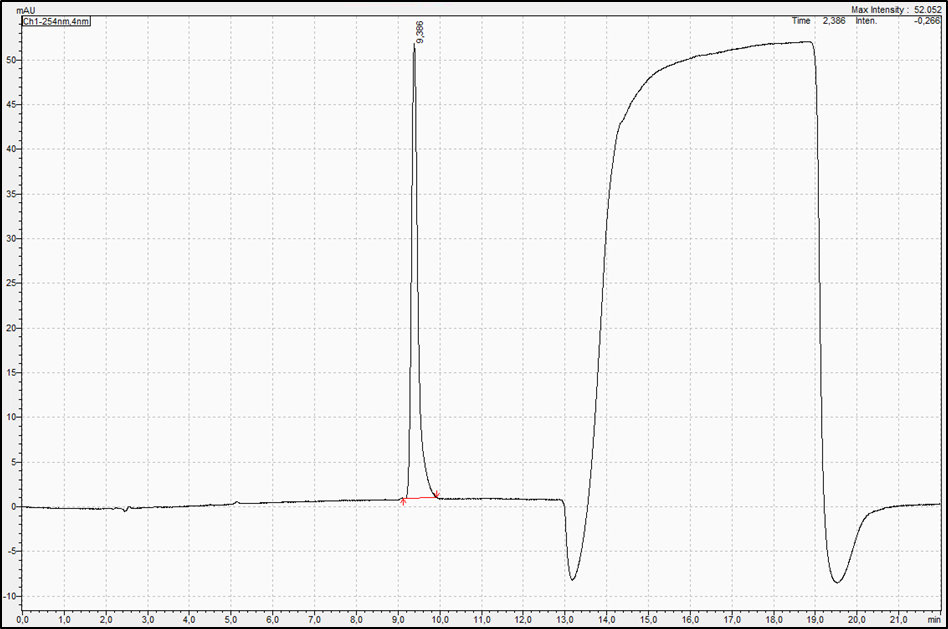


Radio-TLC chromatogram

A Merck silica gel F-254 aluminium plate and ethyl acetate/acetone (1:1, v/v) as eluent were used.


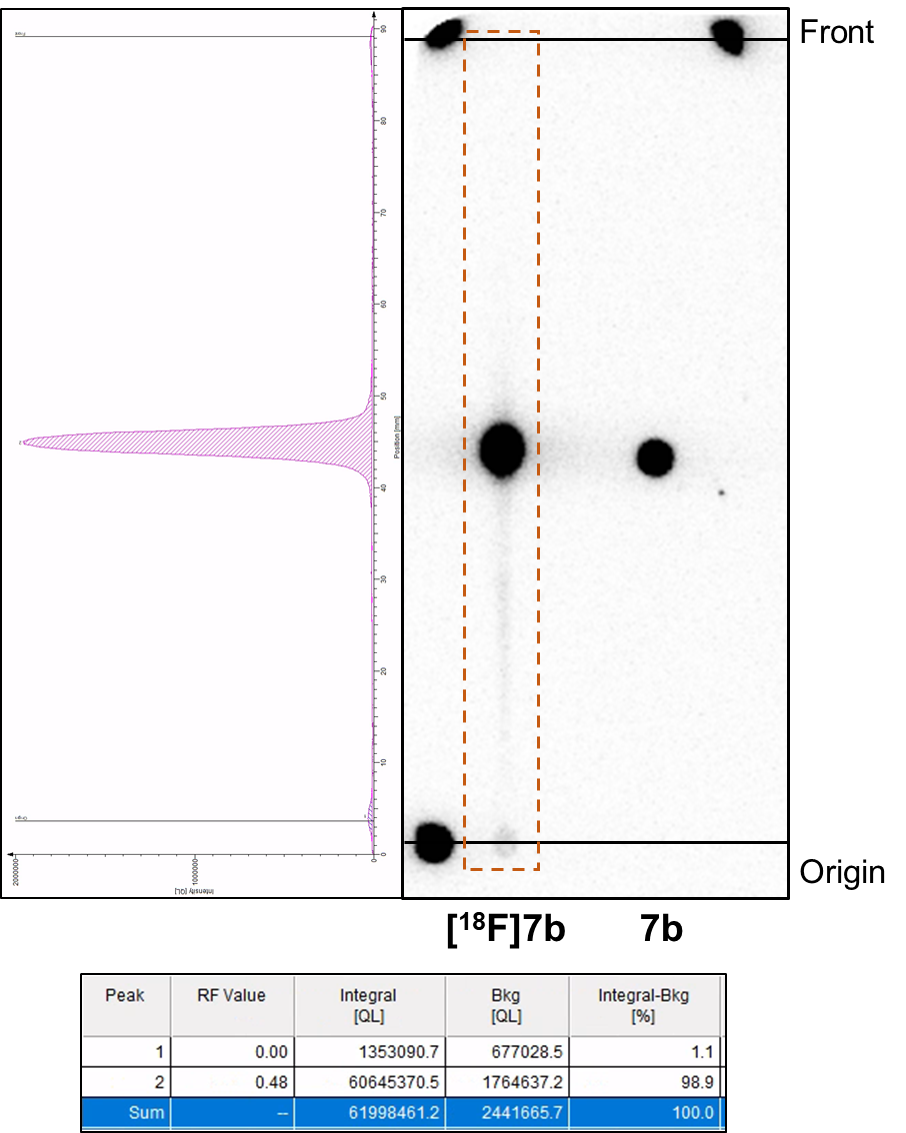


Figure S3: Time course of residual ^18^F-activity and fraction of [^18^F]7b in blood *ex vivo*

Residual activity is expressed as percentage of injected dose (ID) per ml blood.

Figure S4: *Ex vivo* distribution of ^18^F-activity in blood components of a Wistar rat

**A**) Distribution of ^18^F-activity in blood up to 120 min *p.i.* after separating plasma from erythrocytes. **B**) Distribution of ^18^F-activity in plasma up to 120 min *p.i.* after precipitation of plasma proteins (pellet). In both graphs, the sum of fractions is not equal to 100% due to loss of ^18^F-activity during work-up (e.g. binding to pipette tips)

Figure S5: Stability of [^18^F]7b in plasma and erythrocytes after incubation in vitro and in vivo

Radio-HPLC chromatograms obtained for **[^18^F]7b** after incubation under the given conditions.


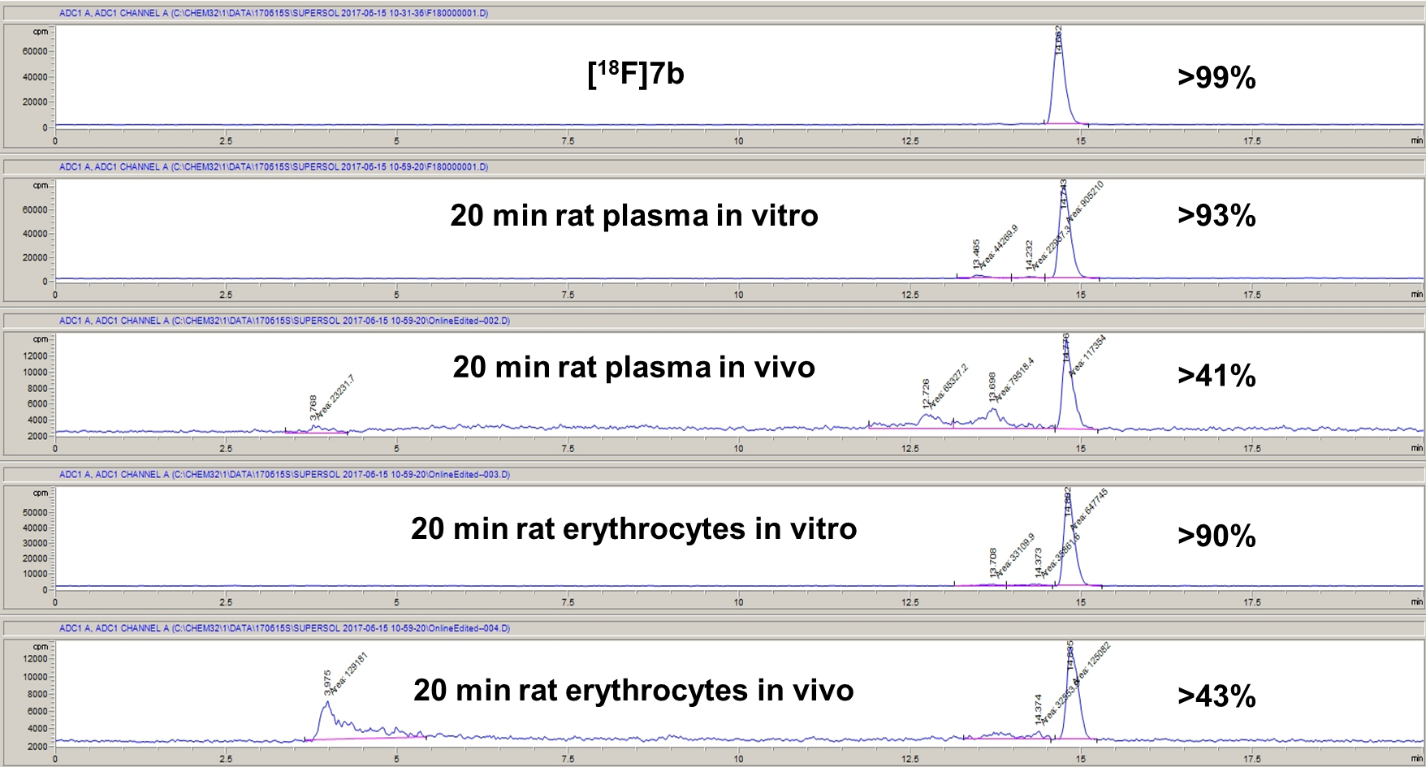


Figure S6: *K*_m_ and V_max_ determination for the degradation of [^18^F]7b by murine liver microsomes

**A**) Time course of residual intact **[^18^F]7b** toward incubation with MLMs at different c.a. formulations as determined by radio-TLC including nonlinear regression according to one-phase decay. **B**) To illustrate the actual turnover of **7b**, the time course of residual intact **7b** (total concentration of **7b** and **[^18^F]7b**) is shown based on the data shown in **A** including nonlinear regression according to one-phase decay. **C**) Initial rates (µM/min) vs. concentration of **7b**. The obtained *k*_obs_ values (in min^-1^) from nonlinear regression of the data shown in **A** were multiplied with the respective total concentrations of **7b** (as given in the legend) to calculate the initial rates (in µM/min) for the degradation of **7b**. These rates were then plotted against the concentration of **7b** to get the common Michaelis-Menten plot and analysis was performed according to the Michaelis-Menten equation to obtain *K*_m_ and V_max_.

Figure S7: Comparison of the profiles of radiometabolites *ex vivo* and toward MLMs

Radio-HPLC chromatograms of **[^18^F]7b** after incubation with murine liver microsomes (MLMs) for 0.5 h and in samples of excretion media taken 2 h after i.v. injection of **[^18^F]7b**.

Figure S8: UPLC-MS/MS analysis of carrier added MLM incubations under oxidative conditions

Summary of metabolites of **7b** identified by UPLC-MS/MS analysis. For metabolites M4 and M5 no potential structures could be derived. The derivation of the structures for M1-4 and M6-8 is based on the UPLC-MS/MS data as shown on the next pages.

Shown are from top to bottom: UV chromatogram, total ion chromatogram (TIC) and extracted ion chromatograms for m/z = 482 (parent **7b**), 498 (mono-hydroxylated metabolites), and 480 (metabolite from hydroxy-defluorination or dehydrogenation).


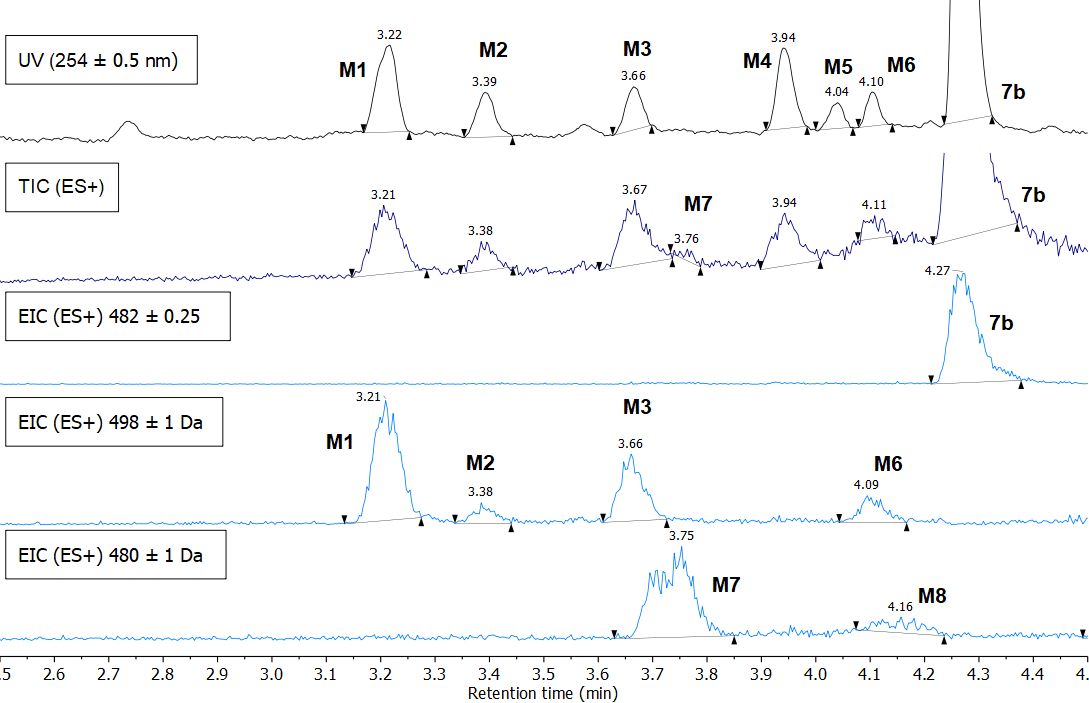

Fragment ion chromatogram of **7b** at two different collision energies (CE) and structural assignment of the observed characteristic fragment ions.


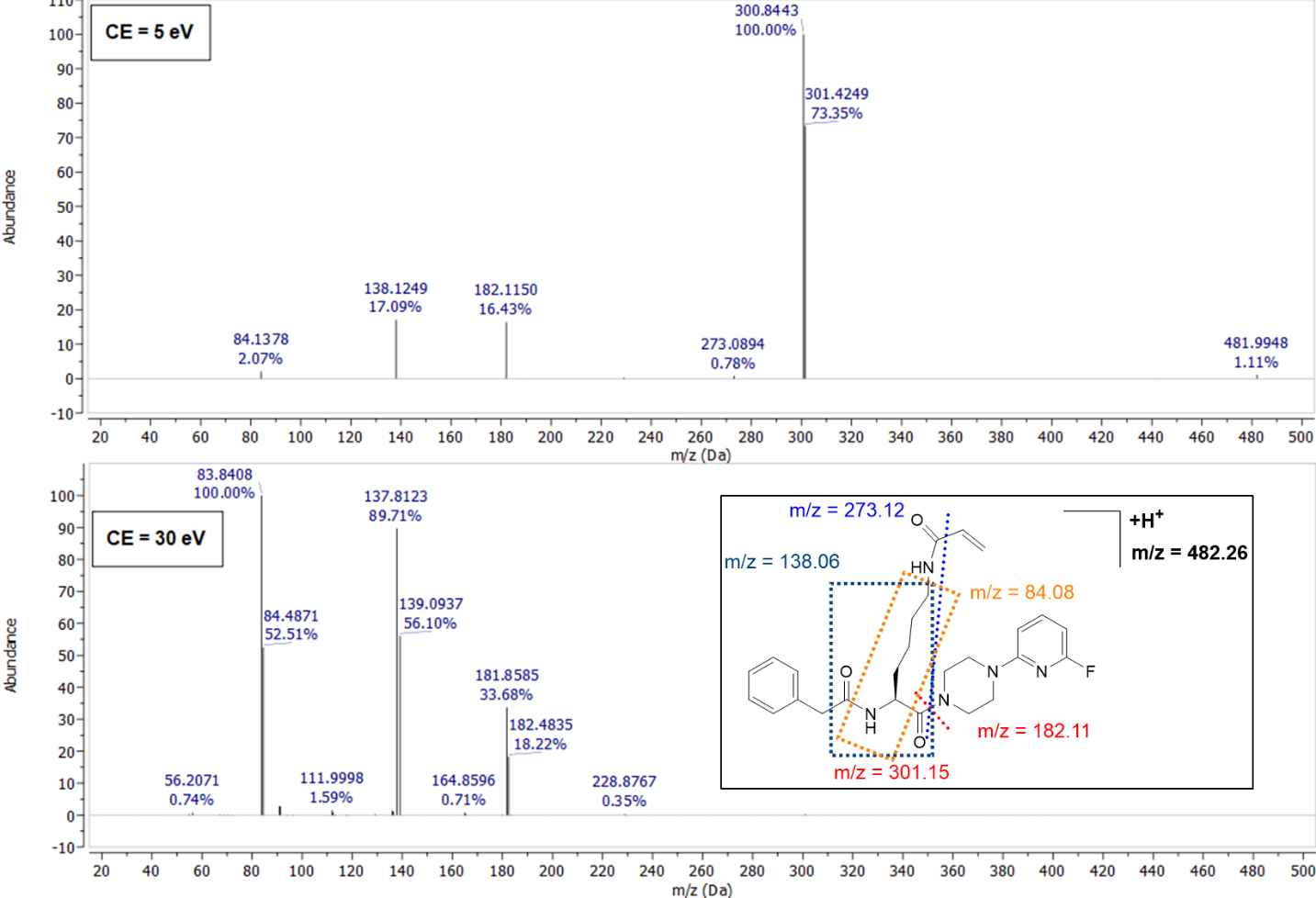


Proposed structures of observed fragment ions

Extracted ion chromatogram for m/z = 498 Da (top left) and fragment ion chromatogram for **M1** (t_R_ = 3.20 min, bottom). The observed fragment ion at m/z = 197.93, which is ≈16 amu higher than the pyridylpiperazine ion from **7b**, indicates hydroxylation at the pyridylpiperazine moiety.


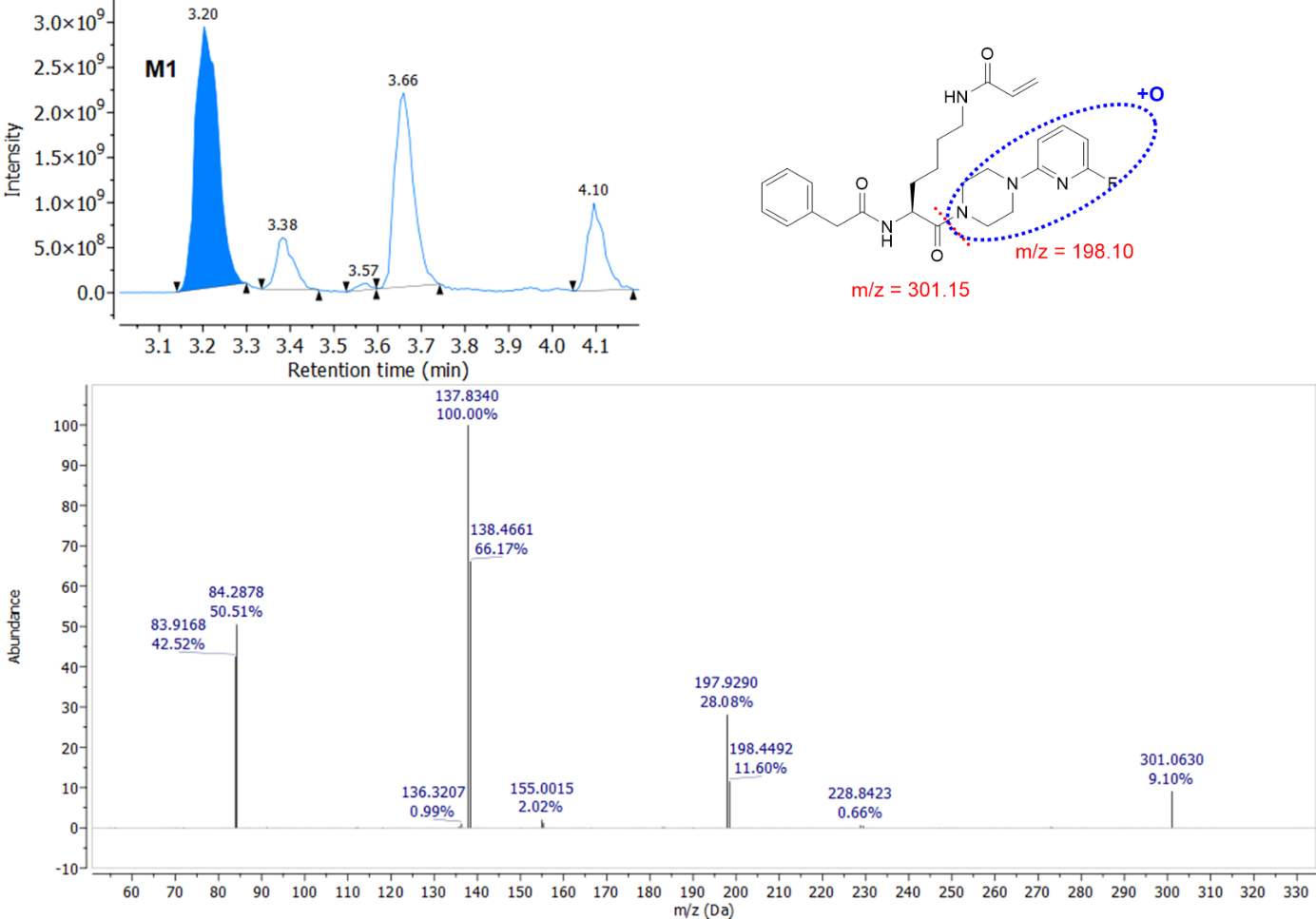


Extracted ion chromatogram for m/z = 498 Da (top left) and fragment ion chromatogram for **M2** (t_R_ = 3.38 min, bottom). The observed fragment ions at m/z = 137.79 and 181.89 are also observed for **7b** and indicate that hydroxylation has not occurred at the pyridylpiperazine moiety and lysine side chain. However, a fragment ion at m/z = 316.82 is visible and indicates hydroxylation at the *N*^α^-phenylacetyl-*N*^ε^-acryloyllysine moiety. We assume that this most likely occurred at the benzyl group, although epoxidation of the acrylamide group is also possible.


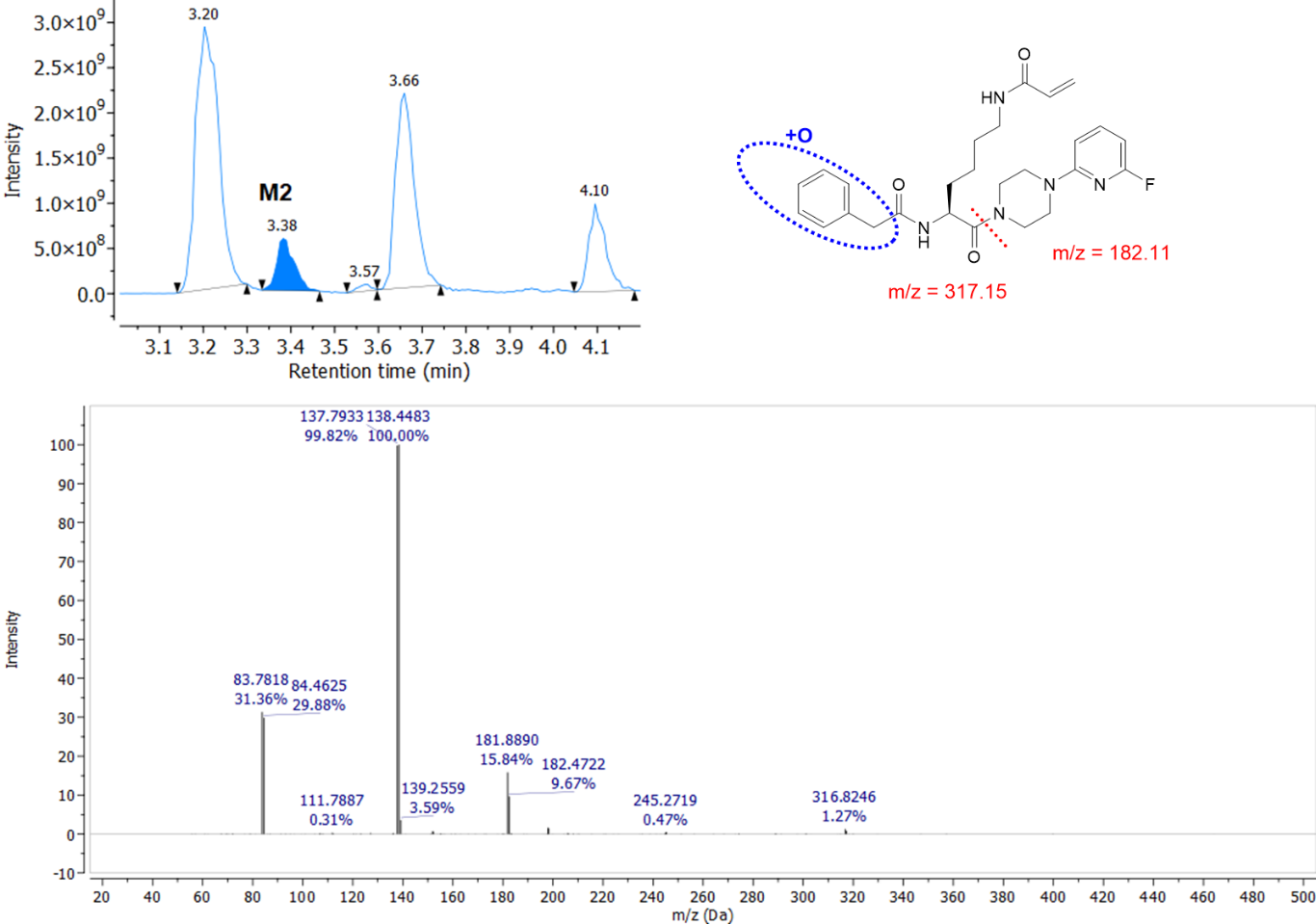


Extracted ion chromatogram for m/z = 498 Da (top left) and fragment ion chromatogram for **M3** (t_R_ = 3.66 min, bottom). The observed fragment ion at m/z = 197.94, which is ≈16 amu higher than the pyridylpiperazine ion from **7b**, indicates hydroxylation at the pyridylpiperazine moiety.


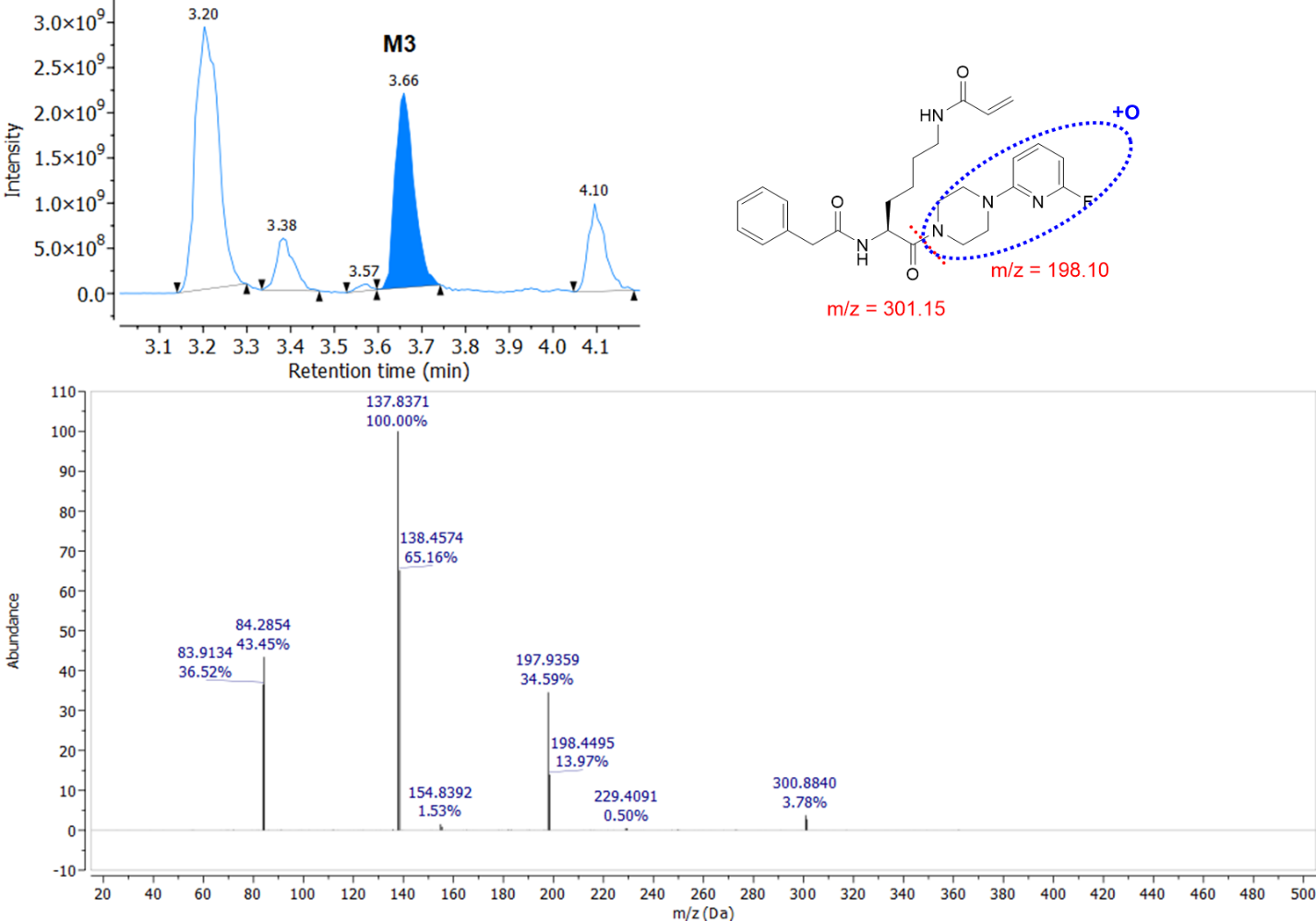


Extracted ion chromatogram for m/z = 498 Da (top left) and fragment ion chromatogram for **M6** (t_R_ = 4.10 min, bottom). The observed fragment ion at m/z = 154.05, which is ≈16 amu greater than the respective ion of **7b** that has the lysine side chain, suggests that hydroxylation occurred at the lysine side chain.


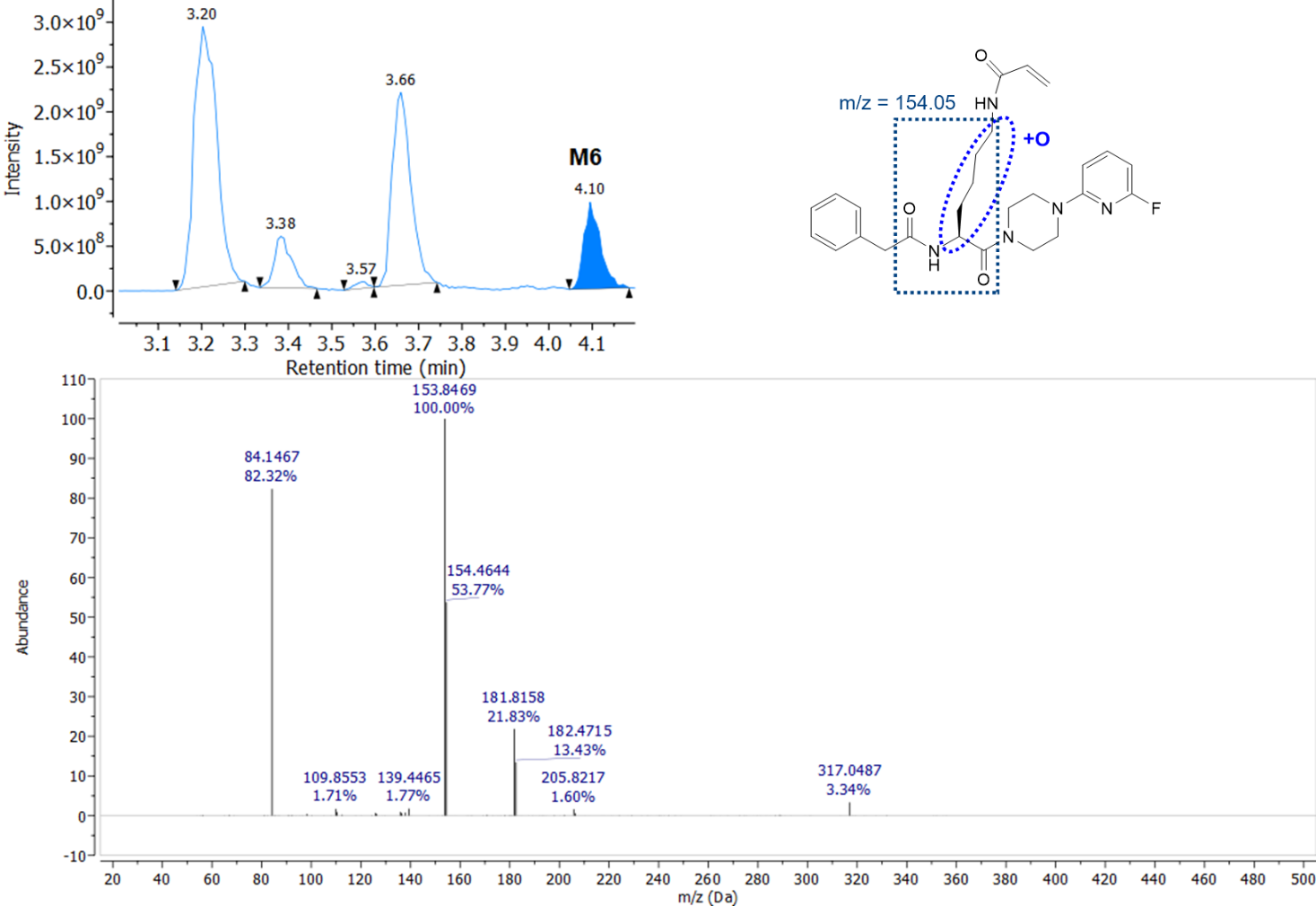


Extracted ion chromatogram for m/z = 480 Da (top left) and fragment ion chromatogram for **M7** (t_R_ = 3.76 min, bottom). The fragment pattern looks different to that observed for the other metabolites and a definite structure of **M7** could not be derived. However, dehydrogenation at the piperazine moiety is supposed.


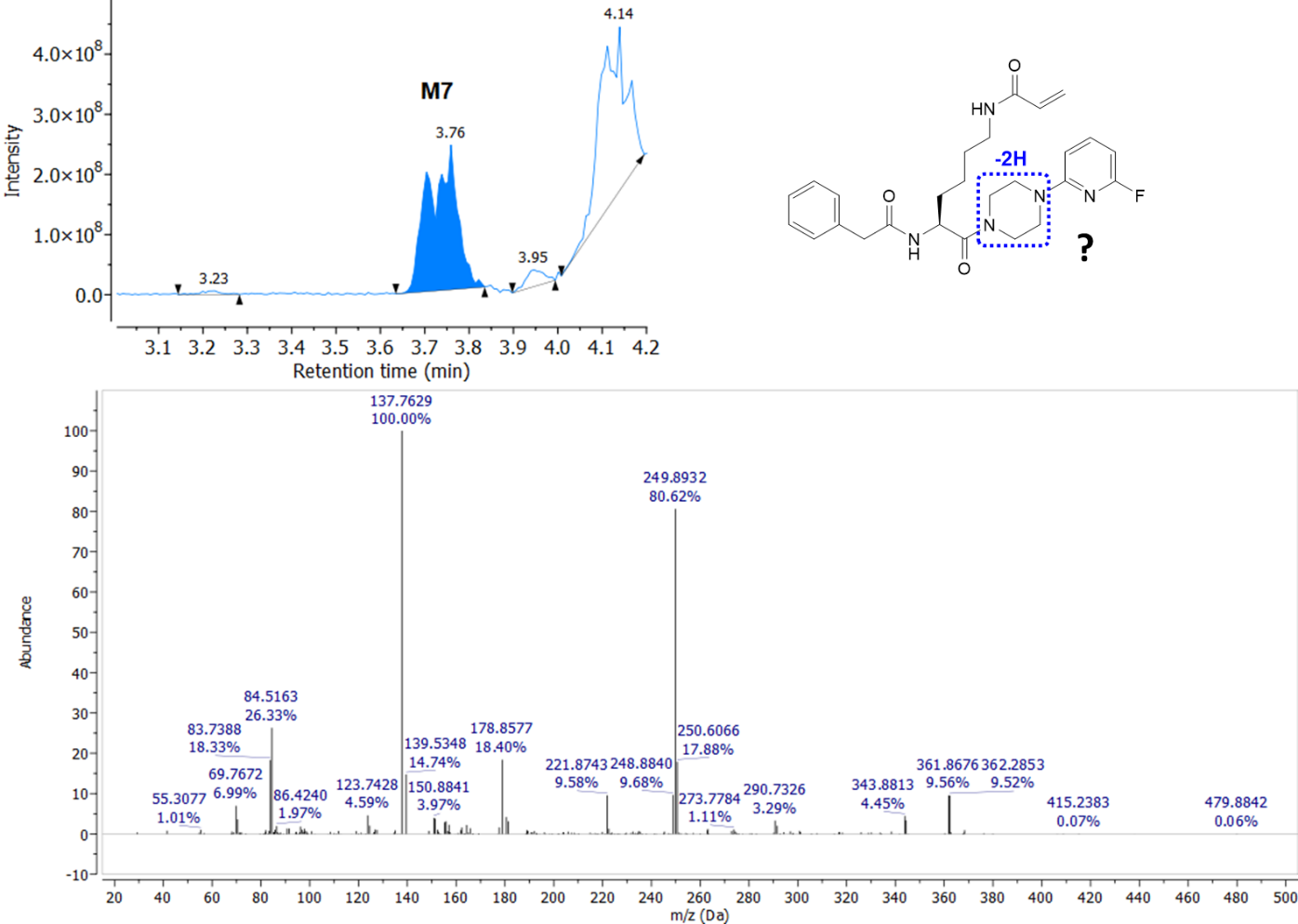


Extracted ion chromatogram for m/z = 480 Da (top left) and fragment ion chromatogram for **M8** (t_R_ = 4.14 min, bottom). The observed fragment ion at m/z = 179.86 indicates a loss of 2 amu within the pyridylpiperazine moiety. Due to the similar retention compared to **7b** (t_R_ = 4.27 min) and the similar fragmentation pattern, the origin of this metabolite from hydroxy-defluorination is supposed.


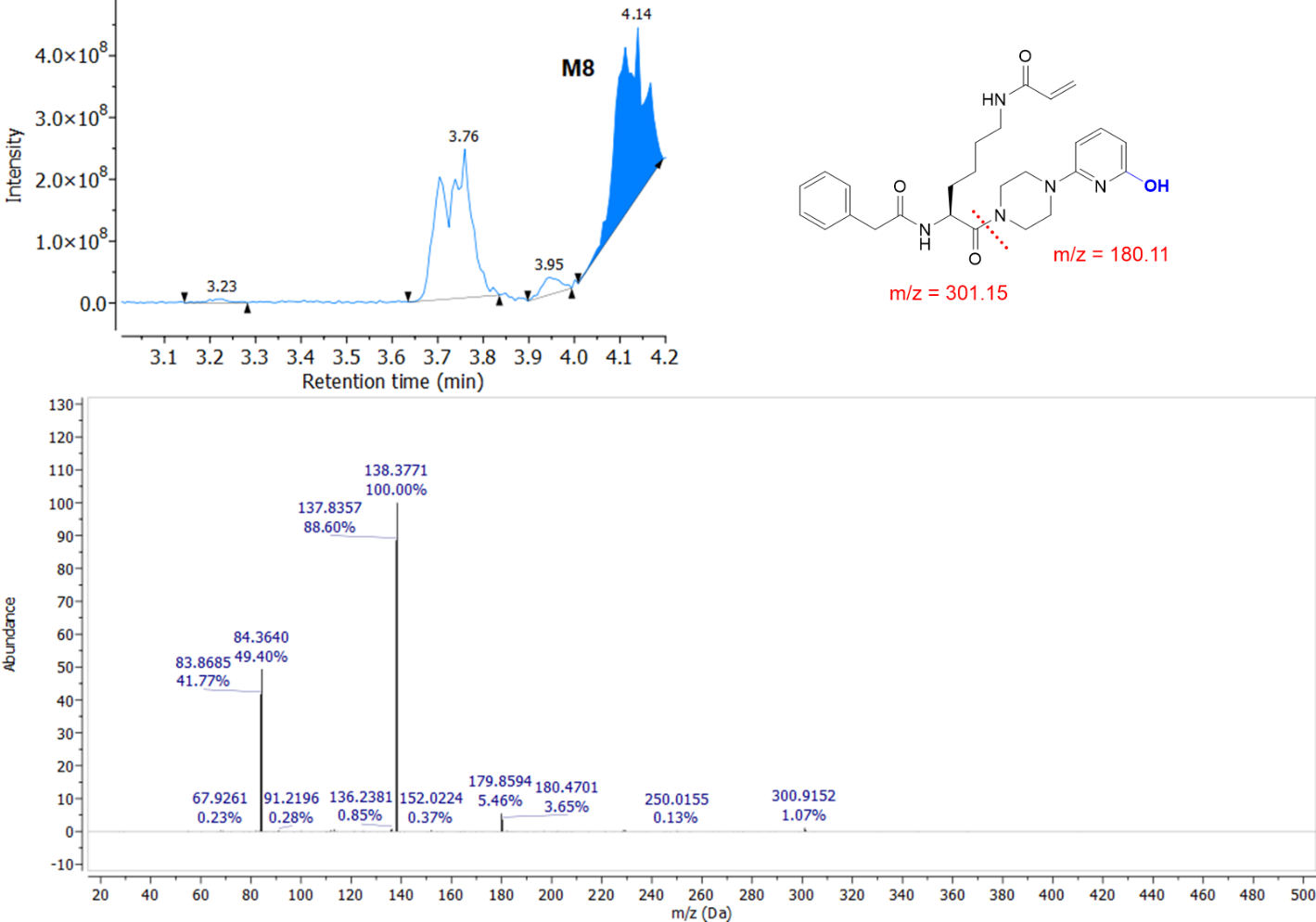


**?**

Figure S9: Comparison of HPLC retention times for 7b-N-oxide and the profile of radiometabolites observed *in vivo*

Radio-HPLC chromatograms of **[^18^F]7b** in samples of excretion media taken 2 h after i.v. injection of **[^18^F]7b**. and UV-HPLC chromatograms (λ=254 nm) of **7b** and **7b-*N*-oxide**. As the order of detectors in the HPLC system is first UV- and then γ-detector, there is a time shift of ≈0.15 min between the UV and radio chromatograms. The two asterisks mark potential peaks in the radio-HPLC chromatograms of intestinal content and urine that could correspond to the respective **[^18^F]7b-*N*-oxide**.

Figure S10: Comparison of HPLC retention times for 7b-*N*-oxide and the profile of radiometabolites observed toward MLM

Exemplary radio-HPLC chromatograms of **[^18^F]7b** after incubation with murine liver microsomes (MLMs) for different time periods and UV-HPLC chromatogram of **7b-*N*-oxide**.

Figure S11: Treatment of [^18^F]7b with MLMs under oxidative condition in the presence of Alamethicin and UDPGA

Radio-TLC of **[^18^F]7b** after incubation with murine liver microsomes (MLMs) for 30 min under oxidation conditions (‘Ox’) and under oxidative / glucuronidation conditions (‘OxGlu’). The TLC spots marked with an asterisk represent the radiometabolite, which was only observed under conditions for glucuronidation. For glucuronidation under oxidative conditions, the same procedure as described in the method section within the main article was followed. In brief, **[^18^F]7b** (100 µL), PBS (60 µL), MLMs (12.5 µL), MgCl_2_ (25 µL of freshly prepared 50 mM solution in PBS, final 5 mM), alamethicin (2.5 µL of 5 mg/mL DMSO stock, final 50 µg/mL) and UDPGA (25 µL of freshly prepared 50 mM solution in PBS) were mixed and warmed for 5 min at 37°C prior to the addition of NADPH (25 µL). For carrier added (CA) incubations, to the solution of **[^18^F]7b** was added **7b** to achieve a final concentration of 50 µM during the MLM incubations.


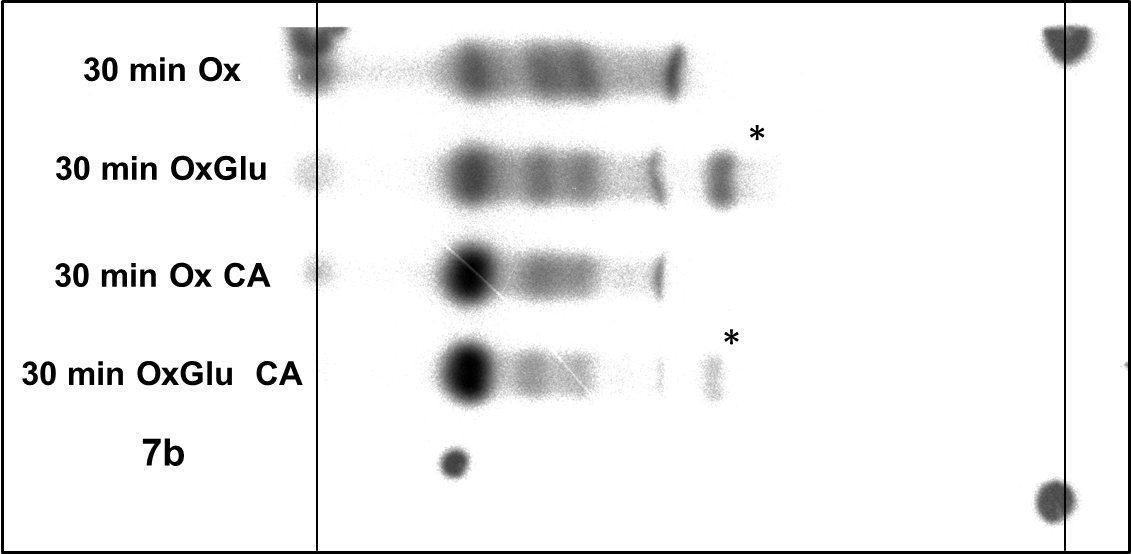


Figure S12: UPLC-MS/MS analysis of carrier added MLM incubations under oxidative/glucoronidation conditions

Shown are from top to bottom: UV chromatogram, total ion chromatogram (TIC) and extracted ion chromatograms for m/z = 482 (parent **7b**) and 674 (mono-glucoronidated metabolite). Other metabolites as obtained from oxidative conditions have not been highlighted for clarity.


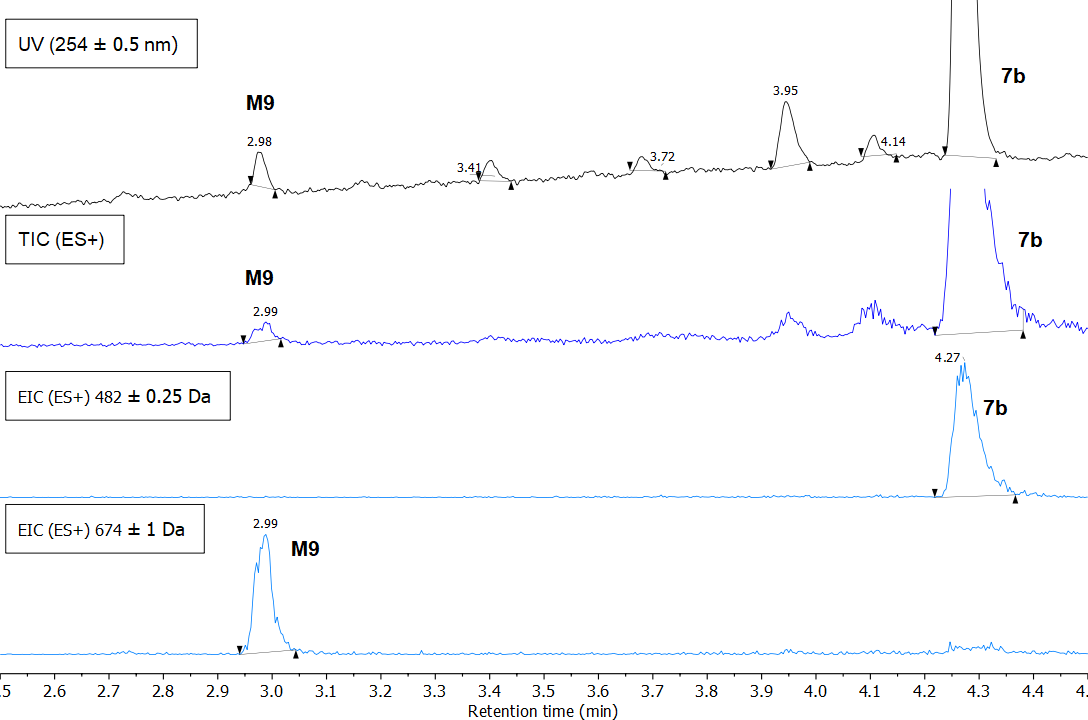

Fragment ion chromatogram for M9. The observed fragment ion at m/z = 374.32, which is ≈192 amu greater than the pyridylpiperazine ion from **7b**, indicates hydroxylation and subsequent glucoronidation at the pyridylpiperazine moiety.


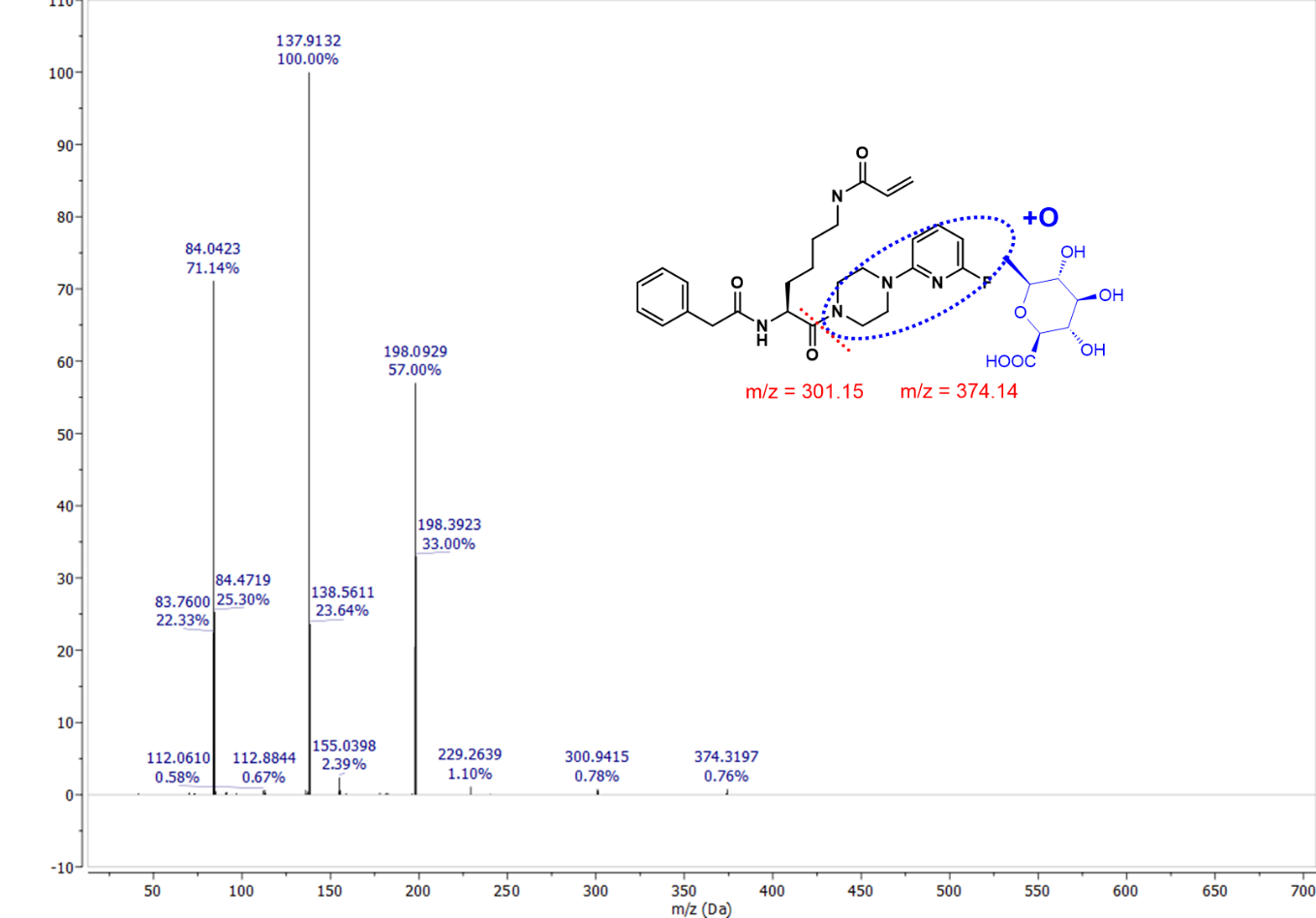


Figure S13: Uptake of [^18^F]7b in different tumor cell lines

Cell were incubated with **[^18^F]7b** alone (0.5 MBq/mL, black curves) and in the presence of either **7b** (10 µM, 0.42 MBq/mL, pink curves) or Verapamil (100 µM, 0.36 MBq/mL, green curves) for 5, 10, 30, and 60 min at 37°C. Data shown are mean values (±SD) of one experiment, which was performed in quadruplicate.

Figure S14: Release of [^18^F]7b from different tumor cell lines

Cell were incubated with **[^18^F]7b** alone and in the presence of Verapamil (100 µM) for 60 min at 37°C. The supernatant was removed and PBS was added. After distinct time points, cell-bound activity and activity in the supernatant was measured. Data shown are mean values (±SD) of one experiment, which was performed in quadruplicate.

Figure S15: Biodistribution of [^18^F]7b in tumor-bearing mice

**A**-**B**) Activity uptake in distinct organs is expressed as activity per g tissue divided by activity per g body weight (i.e. standardized uptake value, SUV) or as percentage of injected dose (%ID; inset) for mice bearing A375-hS100A4 (**A**) or MeWo tumors (**B**). Data shown are mean values (±SD) of 4 mice which received a single injection of **[^18^F]7b** (≈0.5 MBq/animal). BAT and WAT are abbreviations for brown and white adipose tissue. **C**) SUV data only for A375-hS100A4 and MeWo tumor 60 min *p.i.* with and without co-injection of non-radioactive **7b**. Data shown are mean values (±SD) of 4 mice which received a single injection of **[^18^F]7b** (5.23±1.06 MBq/animal for A375-hS100A4 and 6.37±0.15 MBq/animal for MeWo). The molar activities were 52 (n.c.a.) and 0.3 GBq/µmol (c.a., corresponding to 0.3-0.4 mg/kg of **7b**) at time of injection (A_m_ factor of 170).

Figure S16: Hypothetical mechanism of the observed CYP-mediated ^18^F-defluorination of [^18^F]7b assuming plausible hydroxy-defluorination.

The reductase domain, which diverts the two electrons donated by NADPH to the CYP monooxygenase domain, is omitted for clarity. The protoporphyrin IX cofactor is schematically represented by the open circle. For simplicity, the catalytically active high-valent iron-oxo complex is formulated in the formal oxidation state of +5, while in reality this oxidation state is a combination of the oxidation number of +4 for iron and a positive charge delocalized over the protoporphyrin IX tetrapyrrole ring system ([Meunier et al. 2004](#_ENREF_2); [Denisov et al. 2005](#_ENREF_1)).

The Fe(IV/V)-oxo species that is formed during the catalytic cycle electrophilically attacks the 6-position of the pyridine ring. Then, presumably, two electrons are directed from a second equivalent of NADPH through the reductase domain of the CYP monooxygenase to the neighboring C atom of the σ complex. Subsequently, the Fe-O bond is broken and fluoride and the hydroxy-defluorinated product are released. Further investigations are required for the confirmation of this mechanistic proposal.

Scheme S1: Structures of previously reported radiotracers for TGase 2

Table S1: Summary of pharmacokinetic parameters for [^18^F]7b determined in a healthy Wistar rat

|  | HPLC | TLC |
| --- | --- | --- |
| **c_02_ (nM)** | 22.4 | 18.4 |
| ***k*_el_ (min^-1^)** | 0.040 | 0.044 |
| **V_d_ (mL)** | 235 | 287 |
| **V_d_ (mL/kg)** | 1,383 | 1,689 |
| **terminal t_1/2_ (min)** | 17 | 16 |
| **Plasma CL (mL*min^-1^)** | 9.5 | 12.6 |
| **Plasma CL (mL*min^-1^*kg^-1^)** | 56 | 74 |

Pharmacokinetic parameters were calculated based on the blood sampling data from a Wistar rat shown in Figure 3.

Table S2: Summary of pharmacokinetic parameters for [^18^F]7b derived from experiments with murine liver microsomes

|  | **t_1/2_ approach (at 3-4 µM)** | |
| --- | --- | --- |
|  | HPLC | TLC |
| **MLM t_1/2_ (min)^b^** | 5.7 | 7.2 |
| **pred. CL_int_ (mL*min^-1^)^b^** | 15 | 12 |
| **pred. CL_int_ (mL*min^-1^*kg^-1^)^b^** | 772 | 611 |
|  | **V_max_/K_m_ approach** | |
| **V_max_ (µM*mL*min^-1^*mg^-1^)** | 0.37 | |
| ***K*_m_ (µM)** | 1.5 | |
| **V_max_/*K*_m_ (mL*min^-1^*mg^-1^)** | 0.25 | |
| **pred. CL_int_ (mL*min^-1^)** | 32 | |
| **pred. CL_int_ (mL*min^-1^*kg^-1^)** | 1610 | |

^b^Values for the half-life toward murine liver microsomes (MLM) were used for predicting the intrinsic clearance (CL_int_) as described in the Experimental Section.

References for Supporting Information

Denisov IG, Makris TM, Sligar SG, Schlichting I. Structure and chemistry of cytochrome P450. Chem Rev. 2005;105(6):2253-77.

Meunier B, de Visser SP, Shaik S. Mechanism of oxidation reactions catalyzed by cytochrome p450 enzymes. Chem Rev. 2004;104(9):3947-80.
